# 3D‐Printed Bioceramic Scaffolds Reinforced by the In Situ Oriented Growth of Grains for Supercritical Bone Defect Reconstruction

**DOI:** 10.1002/advs.202408459

**Published:** 2024-11-13

**Authors:** Boqing Zhang, Kaixin Wang, Xingyu Gui, Wenzhao Wang, Ping Song, Lina Wu, Likun Guo, Changchun Zhou, Yujiang Fan, Xingdong Zhang

**Affiliations:** ^1^ National Engineering Research Center for Biomaterials Sichuan University 29 Wangjiang Road Chengdu 610064 P. R. China; ^2^ College of Biomedical Engineering Sichuan University 29 Wangjiang Road Chengdu 610064 P. R. China; ^3^ Department of Orthopedics Qilu Hospital of Shandong University 107 Wenhua Road Jinan 250000 P. R. China; ^4^ Department of Orthopedics Orthopedic Research Institute West China Hospital Sichuan University 37 Guoxue Road Chengdu 610041 P. R. China

**Keywords:** bioceramic, grain growth regulation, mechanical enhancement, osteoinduction supercritical bone defect

## Abstract

Porous calcium phosphate ceramics have attracted widespread attention owing to their excellent bioactivity. However, their poor mechanical properties severely limit their clinical applications. Significantly improving the mechanical strength of porous CaP ceramics while maintaining their bioactivity remains a major challenge. To address this issue, calcium sulfate is used to regulate the directional growth of hydroxyapatite grains during ceramic sintering. The in situ oriented grains can not only alleviate the stress concentration but also strengthen the bonding force between the ceramic grain boundaries. Calcium sulfate improves the release of active calcium ions from calcium phosphate ceramics, further enhancing their bioactivity and osteoinductivity in vivo. Transcriptome and proteome sequencing reveals that the in situ whisker‐reinforced ceramics increase the expression of proteins related to calcium ion binding and promote the expression of osteogenesis‐related proteins. In the supercritical bone defect repair model, repair of the defect is achieved within 3 months, with mechanical recovery reaching more than 70% of the autologous bone.

## Graphical abstract

This study induced the in situ oriented grain growth of 3D‐printed porous calcium phosphate ceramic through calcium sulfate, resulting in a mechanically enhanced microstructure, which increased the compressive strength of the porous calcium phosphate ceramic by 5–10 times. Additionally, the release of active calcium ions is enhanced, further optimizing the bioactivity for regenerative repair of supercritical bone defects.

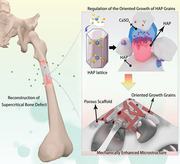

## 1 Introduction

Calcium phosphate (CaP) ceramics, such as hydroxyapatite (HAP), tricalcium phosphate (TCP), and biphasic calcium phosphate (BCP), have been employed as synthetic materials for autologous bone owing to their stable physicochemical properties and excellent biocompatibility, osteoconductivity, and osseointegration.^[^ ^1^, ^2^ ^]^ However, the poor mechanical properties of porous CaP ceramics severely restrict their clinical applications. The compressive strength of bioactive CaP ceramics is often less than 3 MPa, which is far less than that of cortical bone (88–164 MPa).^[^ ^3^, ^4^ ^]^ Therefore, bioactive CaP ceramics are primarily used to repair non‐load‐bearing bone defects or as coating and filling materials.^[^ ^5^, ^6^ ^]^

A highly porous structure can ensure the bioactivity of CaP.^[^ ^7^ ^]^ However, the mechanical strength of the ceramic decreased exponentially with increasing porosity.^[^ ^8^ ^]^ Additionally, the capillary micropores (<20 µm) on the pore wall affect the adsorption of growth factors and other proteins that regulate cytological behaviors.^[^ ^9^, ^10^ ^]^ In ceramic sintering, it is ideal to maintain porous/microporous structures and avoid complete densification caused by high temperature and high pressure; however, these porous/microporous structures are often stress concentration points.^[^ ^11^, ^12^ ^]^ Therefore, improving the mechanical properties of porous CaP while maintaining its bioactivity remains challenging.

Recent studies on the mechanical enhancement of bioactive CaP ceramics mainly focused on two aspects: reducing stress concentration and preventing crack propagation. Combined with finite element mechanical simulations, the shape and porous structure of the ceramic can be optimized to reduce the structural stress concentration, thus enhancing the mechanical strength.^[^ ^13^, ^14^ ^]^ In recent years, with the emergence of ceramic 3D printing technologies, such as digital light projection (DLP), 3D printed CaP ceramics with customizable shapes and internal porous structures have created new possibilities for mechanical optimization and precise repair of bone defects.^[^ ^15^, ^16^, ^17^, ^18^ ^]^ However, this engineering method has certain limitations because of the intrinsic poor mechanical characteristics of ceramic materials. Second‐phase reinforcement is the most widely researched ceramic enhancement method. Its principle is to add a second phase, such as bioinert whiskers, lamellae, fibers, or particles, to the ceramic matrix to slow down crack propagation, thus improving mechanical properties.^[^ ^19^ ^]^ Some materials with high mechanical strength and biological stability, such as alumina, zirconia, titanium dioxide, and magnesium oxide, have been used as reinforcement phases for CaP ceramics.^[^ ^20^, ^21^, ^22^ ^]^ Unfortunately, the externally added second phase does not conglutinate in porous ceramics, resulting in limited mechanical improvement. In addition, the second phase is generally non‐bioactive, which might significantly deteriorate the bioactivity and degradability of CaP ceramics.

Another avenue to enhance the mechanical properties of porous CaP ceramics is through regulating the growth of ceramic grains. In a hydrothermal environment, the HAP grain growth depends on the external environment (pH, temperature, and ions in solution), resulting in different morphologies, such as needle‐like, short rod‐like, and lamellar grains.^[^ ^23^, ^24^ ^]^ Similarly, in solid states, the grain morphology of HAP can be changed by the presence of potassium chloride (KCl), sodium chloride (NaCl), potassium sulfate (K_2_SO_4_), calcium sulfate (CaSO_4_), and calcium fluoride (CaF_2_).^[^ ^25^, ^26^, ^27^, ^28^ ^]^ The binding energies between each crystal face of HAP and these substances affect the crystal growth rate, enabling oriented growth of HAP grains and improving the mechanical properties of the ceramic.^[^ ^29^ ^]^

Therefore, this study aimed to explore a novel research scheme that can simultaneously improve the mechanical properties and bioactivity of CaP. High‐precision porous ceramic green bodies were prepared using a bioink composed of CaSO_4_/HAP via DLP printing technology. CaSO_4_ was chosen because it has been successfully applied in clinical practice, and its biosafety has been widely verified.^[^ ^30^, ^31^ ^]^ During the sintering process, CaSO_4_ regulates the in situ oriented growth of the internal HAP crystals, forming a mechanically enhanced microstructure to reinforce the porous CaP. In addition, CaSO_4_ can modulate the release of active calcium ions (Ca^2+^) from porous CaP, further enhancing the bioactivity of the CaP ceramic.

## 2 Results

### 2.1 Fabrication of In Situ Whisker‐Reinforced Ceramics

**Figure 1:**
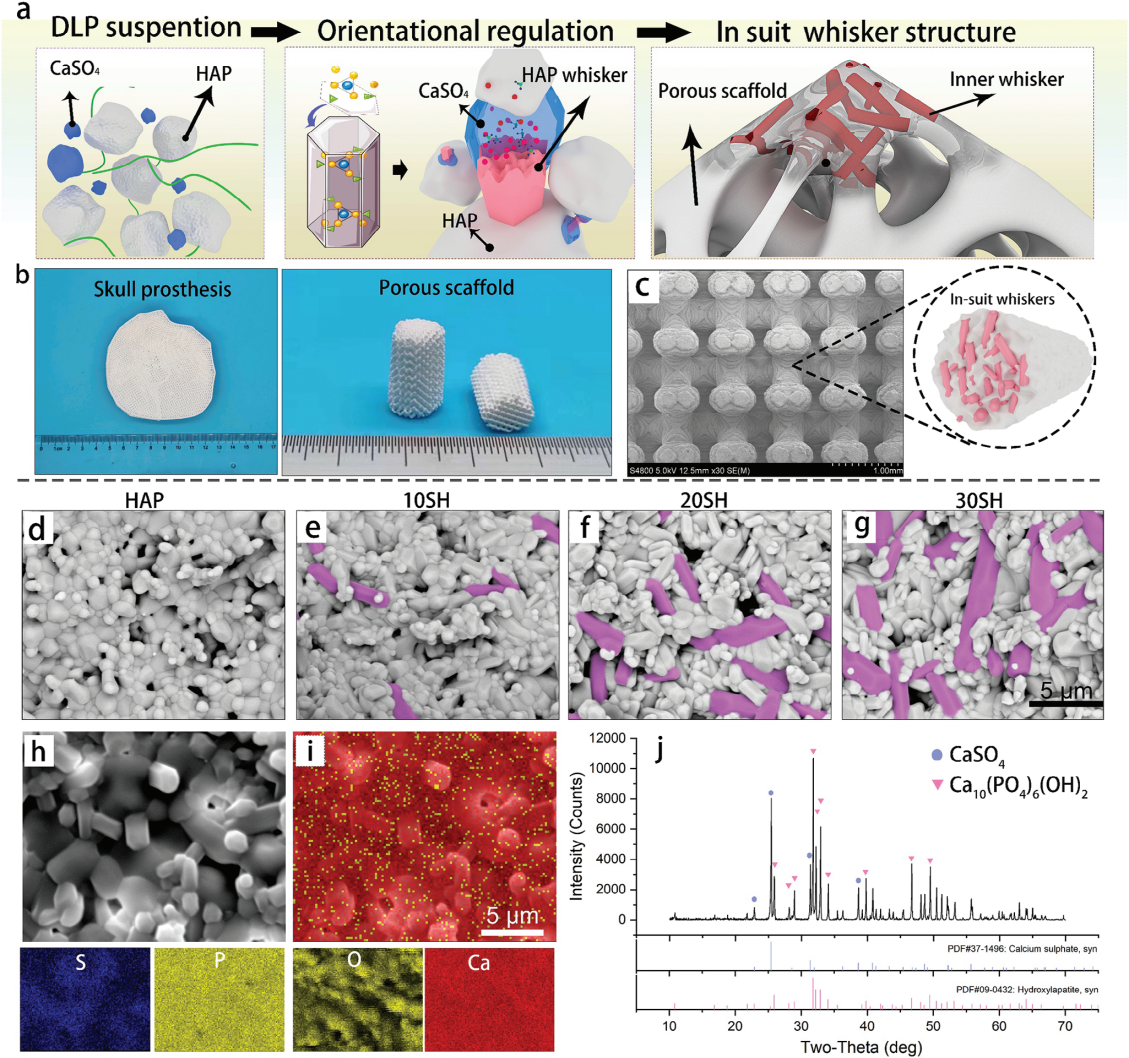
Fabrication of in situ whisker‐reinforced ceramics (IWRC). (a) The regulation process to in situ grow‐oriented HAP grains. CaSO_4_, an active component capable of modulating HAP crystal growth, was incorporated into the printing slurry to circumvent the impact of external whisker addition on the precision of DLP printing. Subsequentially, during the sintering process, the oriented crystals could reinforce the HAP structures. (b,c) Photographs and microstructure of IWRC. The printed scaffolds retain personalized external geometries and high‐precision porous architectures. (d–g) Microstructural morphologies of HAP, 10, 20, and 30 SH. The HAP group consists of oval‐shaped grains and micropores. With the increasing calcium sulfate content, distinct rod‐like whisker structures appear within the ceramic matrix (labeled with purple), and the original picture is shown in Figure S3 (Supporting Information). (h,i) EDS mapping analysis of the 20 SH sample. (j) X‐ray diffraction (XRD) pattern of the IWRC with main characteristic peaks corresponding to those of CaSO_4_ and HAP.

First, a high‐precision ceramic green body was fabricated using the HAP‐CaSO_4_ bioink through DLP technology. In the sintering process, CaSO_4_ could regulate the oriented growth of HAP grains to form an in‐situ whisker structure (**Figure** **1**a). Compared with externally added second phases, CaSO_4_ has less impact on the printing process and printing accuracy. The prepared porous ceramic scaffold is shown in Figure 1b,c, which has a clear outline and porous structure. To observe the in situ oriented growth of the grains, a system composed of 50 wt% CaSO_4_ + 50 wt% HAP (referred to as 50 SH) was first studied. The 3D printed 50 SH ceramic green body was maintained at 900 °C for different times (2, 6, 8, and 10 h) to induce whisker formation and then at 1150 °C for 2 h to complete the sintering, as shown in Figure S1 (Supporting Information). After 2 h at 900 °C, whiskers formed within the HAP matrix with a length of ≈2 µm. After 6 h at 900 °C, a large number of short rods appeared inside the matrix, exhibiting distinct whisker characteristics, with some whiskers reaching a length of 4 µm. After 8 h, the whiskers grew further. When the holding time reached 10 h, the matrix was fully whiskerized, leading to clean prismatic whiskers with diameters of ≈1–2 µm and lengths up to 10 µm. Compared to pure HAP, no significant changes were observed in the matrix morphology, indicating that long‐term heating at 900 °C did not alter the morphology of the original material. These phenomena illustrate that CaSO_4_ can affect the growth of the internal grains at high temperatures; therefore, it is promising to regulate the grain morphology for mechanical optimization. However, the high CaSO_4_ content can degrade the stability of the entire material, and the entire matrix of 50 SH underwent whiskerization and densification. Therefore, it is necessary to optimize the ceramic composition and microstructure.

3D‐printed ceramic green bodies containing 5, 10, 20, and 30 wt% CaSO_4_ in HAP were prepared. After debinding, the samples were maintained at 900 °C for 10 h and subsequently sintered to obtain whiskerized ceramic samples (referred to as 5, 10, 20, and 30 SH respectively), with their microstructures shown in Figure 1d–g. The morphology of 5 SH was similar to that of HAP, and no whisker structures were observed. In 10 SH, distinct short rod‐like whiskers emerged with lengths of ≈3–5 µm and diameters of ≈1 µm. With the increase in CaSO_4_ content, the whisker features were more pronounced. In 30 SH, whiskers grew to 10 µm long and were uniformly distributed in the ceramic and closely combined with the matrix. Energy Dispersive X‐ray Spectroscopy (EDS) line scans were used to analyze the elemental composition of individual whiskers. The whiskers were composed of calcium (Ca), phosphorus (P), and oxygen (O), and no sulfur (S) element was detected (Figure S2, Supporting Information). The atomic molar ratio of Ca to P is ≈1.57, which is close to that of HAP. In the EDS mapping, owing to the presence of CaSO_4_, S appeared in the ceramic matrix with an atomic molar ratio of ≈4.12%, and the atomic molar ratio of Ca to P rose to 2.08. All elements were evenly distributed in the ceramic matrix (Figure 1h,i). In the XRD pattern, the characteristic peaks of both CaSO_4_ and HAP were present without significant shifts, and no other impurity peaks were observed. This indicated that the material was composed solely of CaSO_4_ and HAP crystals (Figure 1j). Combined with the EDS analysis, the generated whiskers were determined to be HAP.

### 2.2 Mechanical Properties

**Figure 2:**
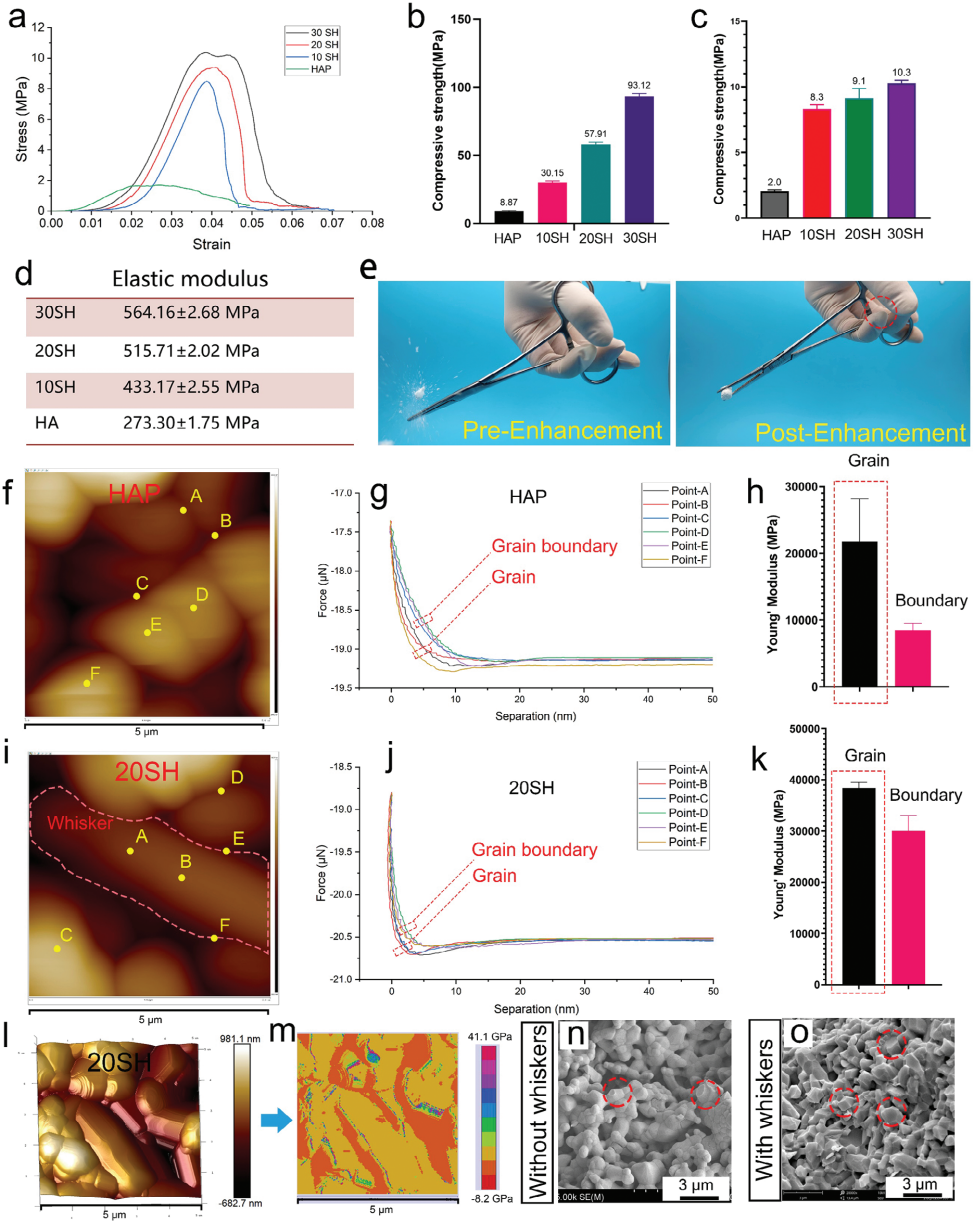
Mechanical properties of IWRC. (a) Stress‐strain curves of the four porous scaffolds. (b,c) Compressive strength of solid and porous ceramic scaffolds. (d) Elastic modulus of porous ceramic scaffolds. (e) Visual comparison of the mechanical reinforcement of the scaffolds demonstrating that the enhanced scaffolds can withstand the bite of surgical forceps, as shown in Movie S1 (Supporting Information). (f,g) Nano‐mechanical testing of HAP, where a probe scanned a 5 × 5 µm area to obtain a surface height map. Six points were selected at both grain boundaries and within grains (f) to measure individual force‐displacement curves (g). The Young's modulus was calculated based on these curves (h). (i,k) Nano‐mechanical properties of 20 SH. (l) A 3D surface height map of 20 SH and the corresponding nano‐mechanical map of the entire area (5 × 5 µm) (m), where the modulus within grains is significantly higher than at the grain boundaries. (n,o) Microscopic morphologies of HAP and 20 SH after fracture, with 20 SH showing numerous trans‐granular fractures.

To understand the effect of the in situ developed whisker structure on the mechanical properties of the ceramic, solid ceramics and porous ceramics with a porosity of 68% were 3D printed for compression testing (**Figure** **2**a–d). The stress‐strain curves of the porous ceramics showed that all scaffolds exhibited brittle fracture, i.e., the stress decreased rapidly after material failure. The in situ developed whiskers significantly strengthened HAP. In the solid pieces (the print model was a solid cylinder), the compressive strength of HAP was 8.87 MPa, whereas that of 30 SH increased by ≈10 times to 93.12 MPa. In porous ceramics, the compressive strength of HAP was only 2 MPa, whereas 30 SH exhibited a strength of 10.3 MPa, which represented an approximate five‐fold enhancement. The elastic moduli of the four materials were calculated based on the stress‐strain curves, revealing that pure HAP,10, 20, and 30 SH had elastic moduli of 273.30 ± 1.7, 433.17 ± 2.55, 515.71 ± 2.02, and 564.16 ± 2.68 MPa, respectively. Owing to the inadequate mechanical strength of porous HAP ceramics, they were prone to fracture during surgical implantation and break into numerous fragments, leading to implant failure. With the reinforcement of in situ grown whiskers, the 20 SH scaffold remained intact when subjected to a compressive force of the hemostatic forceps, whereas the HAP scaffold easily fractured, as illustrated in Figure 2e and Movie S1 (Supporting Information). This demonstrates that the whisker‐reinforced ceramic scaffolds possess the strength required to withstand normal surgical manipulations.

The nanomechanical properties of the materials were further investigated using atomic force microscopy (AFM), in which the force‐displacement curves were measured at three distinct points across the grains and grain boundaries for both the HAP and 20SH samples (Figure 2f–m). The Young's modulus within the crystal was significantly higher than that at the grain boundaries. The nanomechanical mapping of the material confirmed that the forces at the ceramic grain sites were greater than those at the grain boundaries. Within the HAP sample, the Young's modulus within the grains was ≈21.8 GPa, while it was 8.5 GPa at the grain boundaries, yielding a ratio of 0.39. In contrast, in the whisker‐reinforced sample, the modulus values were increased to 38.5 GPa (within the grains) and 30.2 GPa (at the grain boundaries), and the ratio was increased to 0.78. Compared to HAP, the grain boundaries in 20 SH were significantly reinforced, possibly because CaSO_4_ accelerated CaP grain fusion, which also contributes to the mechanical properties of the scaffold. The fracture morphology (Figure 2n,o) indicated that the whisker‐reinforced ceramics exhibited both trans‐granular and intergranular fracture modes, whereas pure HAP ceramics only demonstrated intergranular fractures. Trans‐granular fractures can consume more energy and enhance mechanical strength more effectively.

### 2.3 Mechanical Enhancement Mechanisms

**Figure 3:**
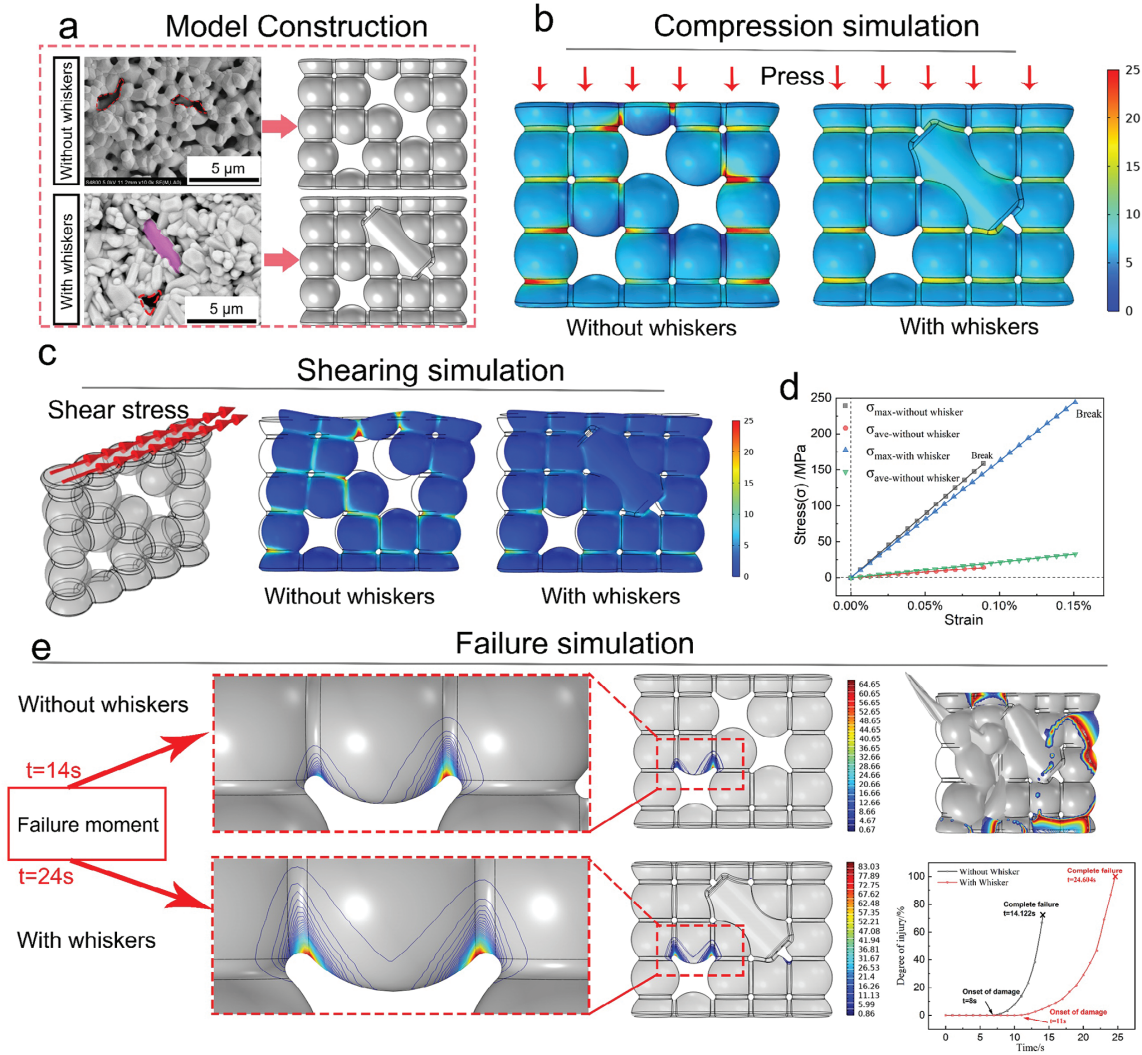
Finite element analysis (FEA) of mechanical enhancement mechanisms. (a) FEA model constructed from Scanning Electron Microscope (SEM) images. The original HAP grains were simplified as spherical shapes, and the cylindrical whiskers were simplified as columnar shapes. (b) Compression simulation of two structures. (c) Shearing simulation of two structures (d) stress‐strain curves. (e–g) Failure simulation of two structures, stress distribution in compression (e), where the group with whiskers had a uniform stress distribution on both sides of the micropores, the morphology after compression failure (f), the time of failure during compression (g), which was 14.12 s for HAP and 24.6 s for the in situ whisker‐reinforced scaffold.

To further understand the mechanism of in situ whisker reinforcement, we designed a simplified model of a 2D monolayer structure based on the microstructural characteristics of the ceramics. The original HAP grains were simplified to spherical shapes, and the cylindrical whiskers were simplified to columnar shapes (**Figure** **3**a). Using static analysis, a compressive stress of 2 MPa was applied to the surface. The calculated Mises stress for the structure without whiskers was 60.138 MPa, whereas the Mises stress for the structure with whiskers decreased to 18.643 MPa. The compressive stress of the scaffold without and with whiskers are 30.989 and 9.173 MPa, respectively. The presence of whiskers significantly reduced the stress concentration under the same conditions. Furthermore, the stress contour plots showed that the whisker structure effectively alleviated the stress concentration at the grain boundaries, regardless of whether the pores were filled. A shear stress of 2 MPa was applied to the upper surface of the scaffold. As observed from Figure 3c, the presence of whiskers significantly improved the deformation resistance of the scaffold, and the average Mises stress decreased from 7.38 MPa for the scaffold without whiskers to 5.29 MPa for the scaffold with whiskers. The stress contour plots showed significant stress concentration in the micropores of the scaffold without whiskers.

We employed a transient model to simulate the material failure during compression. The scaffold base was fixed, and a continuous deformation of 0.5 µm s^−1^ was applied at the top with a time step of 1 s (Figure 3e). In the whisker‐reinforced scaffold, the stress was evenly distributed on both sides of the pores, effectively delaying material failure. The in situ whisker‐reinforced scaffold started to be damaged from the 11th second and completely failed at 24.6 s. However, in the scaffold without whiskers, the stress was mainly concentrated on one side of the pore, and the damage started in the 8th second, with the scaffold completely failing within 14 s. Bioceramics, unlike structural ceramics, require abundant micropores to ensure biocompatibility. However, in terms of the mechanical structure, the edges of these micropores often become stress concentration points to initiate cracks, severely compromising the mechanical performance of the scaffold. According to our stress simulation results, the columnar whisker structure within the ceramic matrix effectively alleviated the stress concentration, delaying the occurrence of damage and improving the mechanical performance of the scaffold. Additionally, when cracks appeared, whiskers effectively impeded crack propagation, thereby enhancing the mechanical performance of the ceramics.

### 2.4 Proliferation and Differentiation

**Figure 4:**
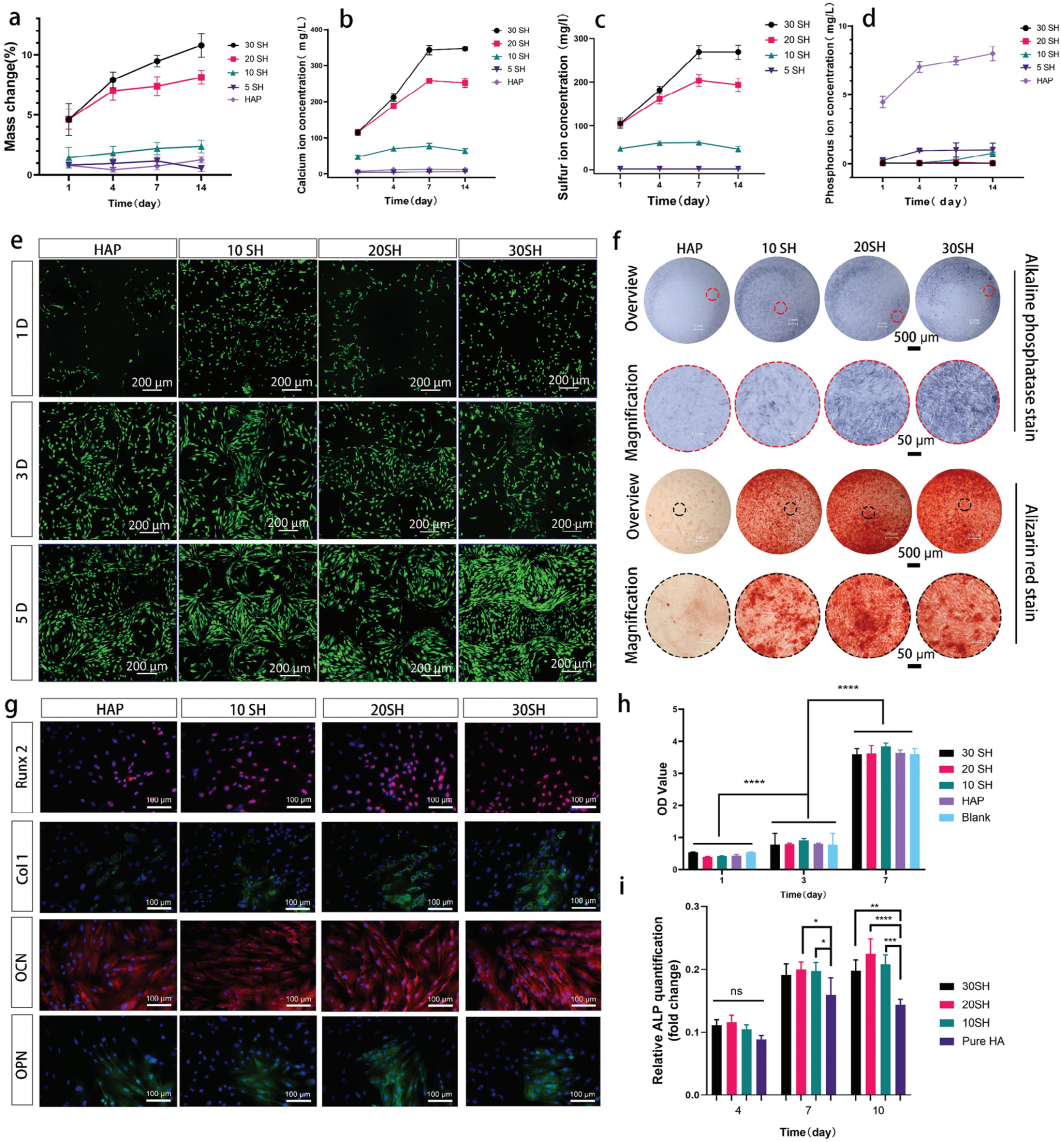
In vitro evaluation of 3D‐printed IWRC with BMSCs. (a) In vitro degradation rate of IWRC. (b–d) The ion release rate of calcium, phosphorus, and sulfur ions. (e) Top‐down view, laser‐scanning confocal reconstructions of live (green) and dead (red) stains on days 1, 3, and 5. (f) Alkaline phosphatase (ALP) staining 10 days after osteoinductive culture with BMSCs, where dark blue is positive expression of ALP. Alizarin red staining 14 days after osteoinductive culture with BMSCs, where red spots were calcium nodules. (g) Immunofluorescence staining 14 days after osteoinductive culture with BMSCs, nucleus (blue), Runx2 (red), Col1 (green), OCN (red), and OPN (green). (h) OD values of the BMSCs in the culture for 1, 3, and 7 days by the CCK‐8 kit (*n* = 6). (i) Semi‐quantitative analysis of ALP on days 4, 7, and 10 (*n* = 3) (^*^ *p* < 0.05, ^**^ *p* < 0.01, ^***^ *p* < 0.001, and ^****^ *p* < 0.0001, ns, not significant).

The composition and degradation products of in situ whisker‐reinforced ceramics (IWRC) are vital for regulating its biocompatibility and biological functions. HAP is a stable phase of CaP with a slow degradation rate in the body. However, CaSO_4_ degrades more rapidly and does not match new bone growth. The addition of CaSO_4_ is expected to improve the degradation rate of HAP ceramics. As shown in **Figure** **4**a, the degradation rate of the IWRC was proportional to the content of CaSO_4_. The mass of HAP remained stable, whereas the mass of 30 SH continuously decreased and reached a mass change of ≈10% on the 14th day. The dissolution rate of CaSO_4_ in water was significantly higher than that of HAP, increasing the release of Ca^2+^ from the ceramic. The 5 SH and HAP scaffolds exhibited consistently low Ca^2+^ release throughout the testing period. The release rates of 20 and 30 SH were significantly higher than the rest, reaching saturation on the 7th day. The saturation concentrations for 20 and 30 SH were ≈230 and 320 mg L^−1^, respectively (Figure 4b). The release pattern of S was similar to that of Ca^2+^, reaching saturation on the 7th day. The saturation concentrations of sulfate ions for 20 and 30 SH were ≈190 and 230 mg L^−1^, respectively (Figure 4c), indicating that the initial release of Ca^2+^ was mainly attributed to CaSO_4_. However, in practical applications, once bone damage occurs, hypoxia and inflammatory responses could cause the pH at the defect site to shift from weakly alkaline to acidic. Additionally, following the defect, osteoclasts became activated and adhered to the damaged area, forming resorption cavities. The osteoclasts secreted hydrogen ions (H+) into these cavities through proton pumps, leading to an acidic environment within the cavities. This acidic microenvironment accelerated the degradation of calcium sulfate/hydroxyapatite scaffolds and further promoted the release of minerals such as calcium and phosphorus.

In vitro biocompatibility assessments of the whisker‐reinforced and HAP scaffolds were conducted using bone marrow stromal cells (BMSCs). The CCK‐8 assay revealed significant cell proliferation on all scaffolds. The optical density (OD) values for 30, 20, and 10 SH at each testing interval were similar to those of the positive blank control and HAP. This indicated that the addition of 10%–30 wt.% CaSO_4_ did not exert cytotoxic effects on the stem cells (Figure 4h). The BMSC proliferations on the four ceramic scaffolds were further compared using laser scanning confocal microscopy (Figure 4e). On day 1 post‐seeding, the cell count was low, and most cells showed spherical shapes without spreading. By day 3, the cells had spread, exhibiting spindle shapes with elongated pseudopodia. The cell count substantially increased across all four materials, and the morphology of the scaffolds began to emerge. Sparse dead cells were observed in 30 SH due to normal cellular apoptosis. By day 5, the cell numbers were further augmented, and cell death was reduced.

After seeding BMSCs onto the scaffold for osteogenic induction, the effect of the IWRC on the osteogenic differentiation of BMSCs was monitored. Alkaline phosphatase (ALP), which induces early osteogenic differentiation, was assessed on day 10 by ALP staining. For 30 and 20 SH, dark and uniform ALP staining was observed, indicating strong ALP expression (Figure 4f). In the coculture within the scaffold, IWRC also possessed high ALP expression (Figure S8, Supporting Information). At later stages of stem cell osteogenic differentiation, calcium ions precipitated as calcium salts to form “bone nodules (calcium nodules).” On day 14 of induction, Alizarin red staining was used to visualize the calcium nodules within the BMSCs. 30 and 20 SH presented deeper colors in larger areas than the other groups, suggesting a higher density of calcium nodules, as shown in the high‐magnification images (Figure 4f). Although the number of calcium nodules in 10 SH was lower than that in 20 and 30 SH, it was significantly higher than that in HAP. Furthermore, several osteogenesis‐related proteins were tested by immunofluorescence (Figure 4g). Runx2, which served as an essential transcription factor for stimulating osteogenesis, showed higher expression in 20 and 30 SH. Stronger fluorescence intensities were also detected in 20 and 30 SH for collagen1 (Col1), a marker in pre‐osteoblasts and mature osteoblasts during BMSC osteogenic differentiation. Osteocalcin (OCN) and osteobridging protein (OPN), produced at the end of osteoblast differentiation, were highly expressed in 20 and 30 SH. Because the cells were not in direct contact with the scaffolds during these experiments, the material composition emerged as a key factor in facilitating osteogenic differentiation.

### 2.5 Transcriptomic Analysis of the IWRC for Osteogenic Differentiation

**Figure 5:**
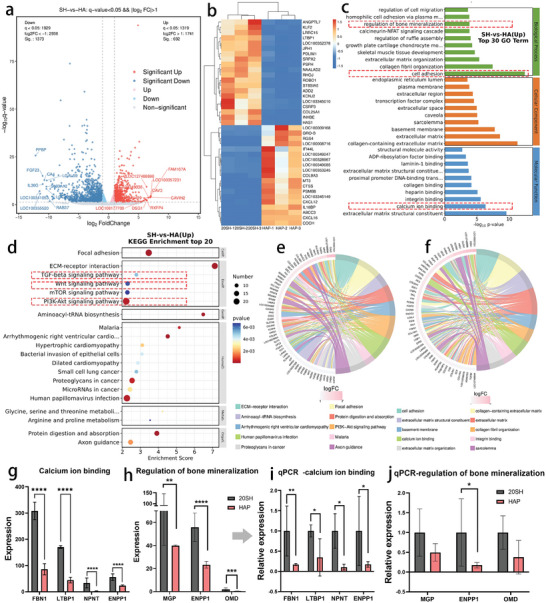
Transcriptomic analysis for BMSCs osteogenic differentiation. (a) Volcano plot of DEGs, grey represents genes without significant differential expression, red represents significantly upregulated genes, and blue represents significantly downregulated genes. (b) Clustered heatmaps of DEGs showing the top 20 genes that are significantly upregulated and downregulated. (c) GO enrichment analysis results, filtering three categories of GO terms with corresponding PopHits ≥5. The top 10 terms were sorted in descending order based on the ‐log10p value. (e) KEGG enrichment analysis results. (d) Top 20 KEGG pathway enrichment analysis (filtered for pathways with corresponding PopHits ≥5, sorted by ‐log10p value in descending order). (e) Top 10 GO enrichment chord diagram. (f) Top 10 KEGG enrichment chord diagram. (g) Expression of genes associated with calcium ion binding in transcriptome sequencing, consistent with the qPCR results (i) (*n* = 3). (h) Expression of genes related to bone mineralization in transcriptome sequencing, consistent with the qPCR results (j) (*n* = 3) (^*^ *p* < 0.05, ^**^ *p* < 0.01, ^***^ *p* < 0.001, and ^****^ *p* < 0.0001, ns, not significant).

To further investigate the molecular mechanism of osteogenic differentiation, we performed a transcriptomic analysis of BMSCs cultured on HAP and 20 SH for 21 days in the absence of osteogenic growth factors. Differential screening was performed based on the expression of protein‐coding genes, and 2065 differential genes were detected, as shown in **Figure** **5**a. A total of 1373 genes were significantly downregulated and labeled in blue, and 692 genes were significantly upregulated and labeled in red. Genes without significant differences are labeled in grey. The top 20 differentially expressed genes (DEGs) that were upregulated or downregulated are shown in the clustered heatmap (Figure 5b). Compared with those in HAP, the expressions of ANGPTL7, KLF2, and LTBP1 in 20 SH were 21.7, 9.73, and 3.69 folds higher, respectively. Gene Ontology (GO) enrichment analysis was performed on the DEGs in terms of molecular functions, biological processes, and cellular components (Figure 5c). Five genes related to bone mineralization, 39 related to cell adhesion, and 41 related to calcium ion binding were significantly upregulated. After the screening, genes related to calcium ion binding, such as FNN1, LTBP1, NPNT, and ENPP1, were significantly upregulated (Figure 5g); MGP, ENPP1, and OMD, which are related to bone mineralization, were also significantly upregulated (Figure 5h). These results were consistent with the transcriptome data measured by qPCR (Figure 5i,j). The enrichment analysis and chord diagram (Figure 5e) illustrated the top 10 categories with the smallest *p*‐values. The Kyoto Encyclopedia of Genes and Genomes (KEGG) database was used for pathway analysis of the DEGs (Figure 5d), and the enrichment score was represented on the horizontal axis. The results indicated that the PI3K‐ART, TGF‐beta, and Wnt signaling pathways were all effectively activated, with the TGF‐beta signaling pathway exhibiting the highest enrichment score, suggesting its predominant role in promoting osteogenic differentiation.

### 2.6 Ectopic Osteoinduction by IWRC

**Figure 6:**
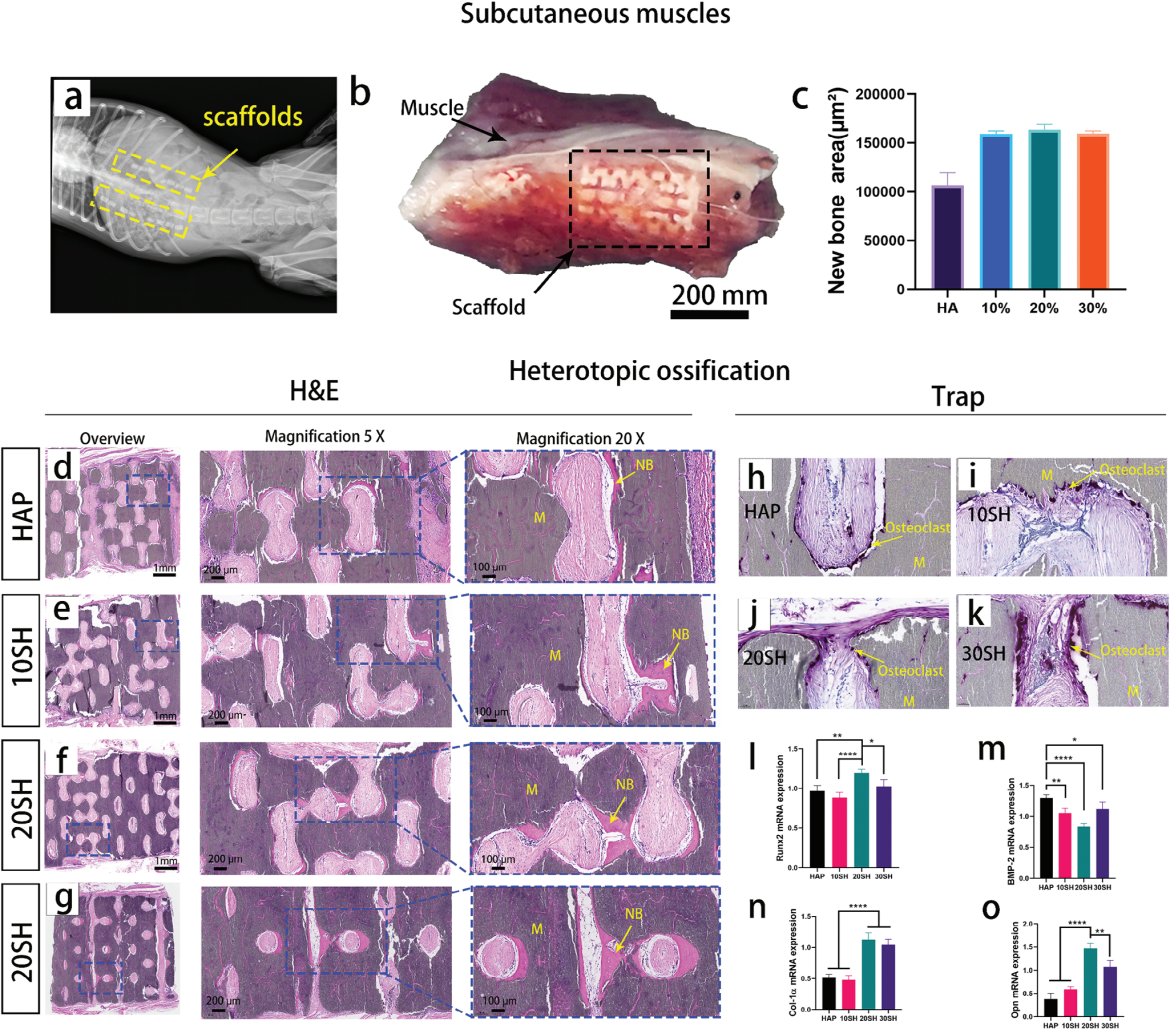
Osteoinduction evaluation in vivo with a beagle dog subcutaneous implant. (a) Representative radiograph illustrating the bilateral placement of the scaffold in the beagle's back muscles. (b) Photograph of the muscular tissue containing scaffolds 3 months after implantation. The placement of scaffolds (black box) is visible. (c) Semi‐quantitative analysis of the new bone tissue area. The calculations were based on histological H&E staining. (d–g) Histological H&E staining images for the four materials. The far‐left column presents an overview of the stained tissue sections. Blue boxes indicate the regions that have been magnified. Scaffold material is denoted by (M), and newly formed bone tissue (NB) is indicated by yellow arrows. (H–K) Histological images of Trap staining for the four materials to identify osteoclasts within the new bone tissue. The presence of osteoclasts is marked by dark red staining (yellow arrows) on the surface of the material (M) (the overall image of Trap staining is shown in Figure S11, Supporting Information). (i–o) Gene expression levels (Runx2, COL‐1α, BMP2, and OPN) of osteogenic‐relevant transcripts in the scaffolds 4 months after implantation (*n* = 3) (^*^ *p* < 0.05, ^**^ *p* < 0.01, ^***^ *p* < 0.001, and ^****^ *p* < 0.0001).

Ectopic ossification is a critical criterion for evaluating the osteoinductive potential of materials. To assess the bone repair capabilities of the IWRC, we implanted four groups of scaffolds (30 SH, 20 SH, 10 SH, and HAP) into the paravertebral muscles of beagles for 3 months to observe the osteogenic phenomena. The relative position of the implant within the canine body is depicted in **Figure** **6**a. Three months post‐implantation, the materials were retrieved from the muscle tissue. The scaffolds had integrated tightly with the surrounding musculature, and tissues grew into the scaffold interiors without evidence of fibrous encapsulation (Figure 6b). Further histological analysis with Hematoxylin and Eosin (H&E) staining revealed that soft tissues proliferated throughout the porous structures of all four scaffolds, with dense, deep red bone tissue detected on the scaffold walls. In HAP, new‐bone tissues showed elongated shapes along the inner porous walls with a thickness of ≈20 µm (Figure 6d). In the whisker‐reinforced groups, new‐bone tissues grew into sheets with thicknesses exceeding 100 µm in certain areas. In particular, contiguous bone tissue was observed within 20 SH (Figure 6e–g). Semi‐quantitative analysis using the ImageJ software showed a significantly greater volume in the whisker‐reinforced group than in the HAP (Figure 6c). This demonstrates that the in situ whisker‐reinforced ceramics retain their osteoinductive properties, outperform HAP ceramics, and show potential for further applications. Tartrate‐resistant acid phosphatase (Trap) staining revealed that osteoclasts emerged within the four groups of materials, adhering closely to the surface of the materials. This indicated that the induced bone tissue had self‐remodeling functions (Figure 6h–k). Following a clear observation of osteoinduction, we employed polymerase chain reaction (PCR) techniques to analyze the expression levels of osteogenesis‐related genes in the four materials (Figure 6l–o). The Runx2 mRNA levels and OPN expression in 20 SH were significantly higher than those in the other three groups. The expression of COL‐1α was also the highest in 20 and 30 SH. Immunohistochemical staining also indicated that IWRC promoted the expression of genes related to osteogenesis and blood vessel generation, as shown in Figure S10 (Supporting Information).

### 2.7 Proteomic Quantitative Analysis of Osteoinductivity in Muscle Tissue

**Figure 7:**
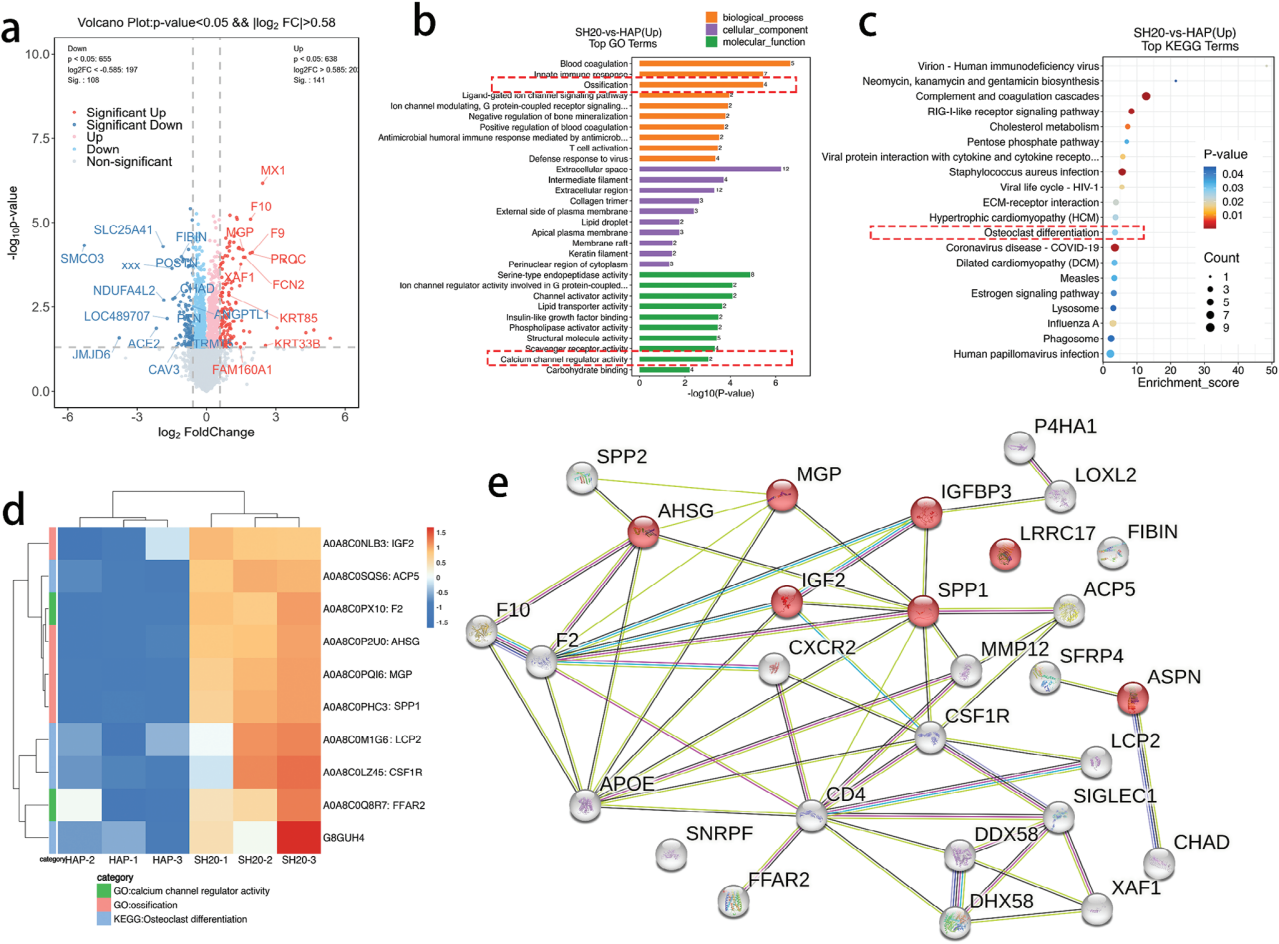
Proteomic quantitative analysis of osteoinductivity in muscle tissue. (a) Volcano plot of differential proteins. The horizontal axis represents log_2_ (fold change). Grey points represent proteins with a *p*‐value ≥ 0.05. (b)Top 30 GO enrichment analysis bar chart. The horizontal axis shows the ‐log10p values, while the vertical axis lists GO term names. (c) Top 20 KEGG enrichment analysis bubble chart. The horizontal axis represents the enrichment score, while the vertical axis shows the information of the top 20 pathways. (d) Cluster heatmap of osteogenesis‐related proteins, selected through GO enrichment and KEGG enrichment analyses. (e) Protein‐protein interaction network with high connectivity, where the proteins related to ossification are marked in red.

To further elucidate the mechanism underlying the enhanced osteoinductivity of IWRC, we conducted quantitative proteomic analysis on the subcutaneously implanted scaffolds after 1 month. At this stage, no significant bone tissue was observed within the material, facilitating the investigation of the mechanisms involved in material‐induced osteogenesis. **Figure** **7**a displays the differentially expressed proteins in the HAP and 20 SH scaffolds, with 141 proteins significantly upregulated and 108 proteins significantly downregulated. Enrichment analysis of the differential proteins, using the GO database, revealed that the 20 SH exhibited significant upregulation in the expression of ossification‐related proteins, such as IGF2, AHSG, MGP, and SPP1. Moreover, the expression of proteins associated with calcium channel regulation was upregulated in 20 SH, with the expression levels of F2 and FFAR2 being 2.02‐fold and 1.53‐fold higher than those in HAP. Figure 6c shows the combined data from the KEGG database, indicating that 20 SH significantly promoted the expression of osteoclast differentiation‐related proteins, including G8GUH4, ACP5, CSF1R, and LCP2. The differences in the expression of these proteins between the two groups are presented in the clustering heat map (Figure 7d). The upregulation of these proteins can promote the development of bone tissues. By selecting related species in the STRING database, we analyzed the interaction relationships of differentially expressed proteins and identified the top 25 proteins by connectivity. After recalculating their connectivity and constructing a network diagram (Figure 7e), it was evident that IWRC may promote osteoinductivity through the networked expression of osteogenesis‐related proteins, such as AHSG, ACP5, IGF2, SPP1, and MGP. Among these, calcium ion‐related proteins, such as F2 and FFAR2, synergistically regulate the expression of osteogenesis‐related proteins.

### 2.8 IWRC Efficacy in Supercritical Bone Defect Regeneration

**Figure 8:**
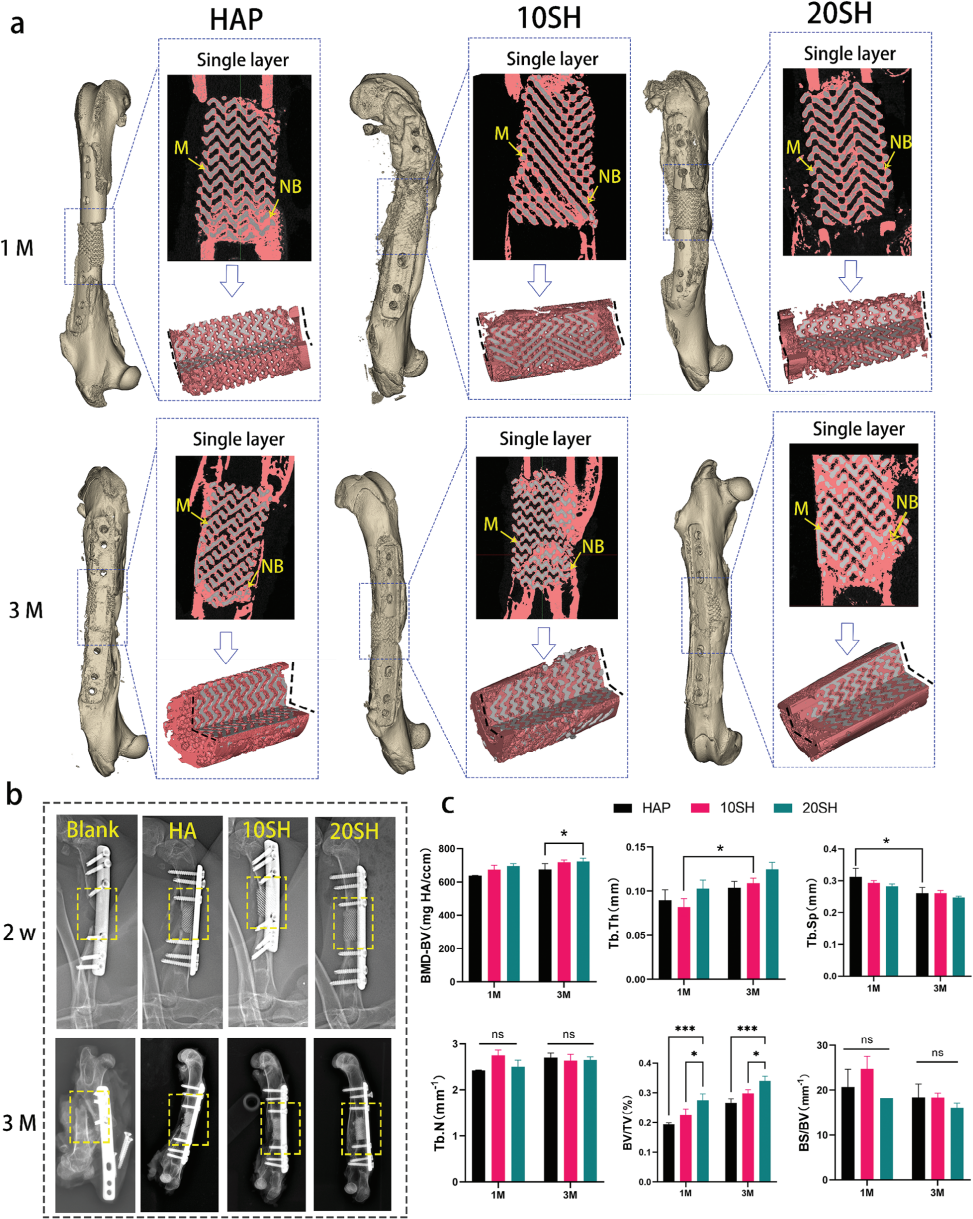
Radiological assessment of bone repair at weight‐bearing sites. (a) Computed tomography (CT) evaluation of bone tissue regeneration within different materials, including post‐repair 3D reconstructions, single‐layer scan images (M: scaffold material, NB: new bone tissue), and quarter‐sectional views of the new bone tissue (red represents bone tissue, and grey represents scaffold material). (b) X‐ray assessments of bone integration status for different scaffolds at 2 weeks and 3 months post‐implantation (yellow outlines denote the experimental sites). (c) MicroCT–rendered parameters of ingrown bone at different time points (*n* = 3) (^*^ *p* < 0.05, ^**^ *p* < 0.01, ^***^ *p* < 0.001, ns, not significant).

To assess the ability of IWRC to promote bone regeneration in preclinical supercritical bone defects, we established a 15 mm truncated bone defect model in rabbit femurs. In this study, the average diameter of the rabbit femur was ≈7 mm, and the defect created in the animal model was twice that size, exceeding the requirement of 1.5 times the critical bone defect threshold. We implanted 10 SH, 20 SH, and HAP scaffolds and observed the growth of new bone. The implantation process and the images obtained after restoration are shown in Figure S13 (Supporting Information). Micro‐computed tomography (micro‐CT) was used to semi‐quantitatively observe the generation of new bone, including the regeneration of new osteal tissue within the implant and the integration of the material with the host bone. **Figure** **8**a presents the post‐repair 3D reconstructions, single‐layer radiographic images, and quarter‐sectional views to compare the overall morphologies of the scaffold and femur at 1‐ and 3‐month post‐implantation. One month after implantation, a clear demarcation between the scaffold and host bone persisted, and a layer of osseous tissue (red) was formed on the internal surface of the scaffold. However, the porous structure of the scaffold was discernible with prominent voids. The proportion of new bone (BV/TV) and density of new bone (BMD‐BV) exhibited a trend of 20 SH > 10 SH > HAP, whereas the trabecular spacing (Tb.Sp) decreased (Figure 8c). Compared to the first month, the new bone tissue significantly increased at 3 months. It can be clearly observed from the micro‐CT images that, in the 10 and 20 SH groups, the new bone tissue has merged into continuous structures and the scaffold has fused with both ends of the bone defect. In conjunction with the semi‐quantitative analysis results, it was found that the proportion of new bone (BV/TV) and new bone density (BMD‐BV) increased with an increase in trabecular thickness (Tb.Th) and a decrease in trabecular spacing (Tb.Sp) compared to the first month. The trends in the volume and density of new bone among the three groups at 3 months were consistent with those observed in the first month, i.e., 20SH > 10SH > HAP.

### 2.9 Clinical Assessment of Bone Healing via X‐ray Imaging

X‐ray imaging is a crucial diagnostic tool for the clinical evaluation of bone healing at weight‐bearing sites. Therefore, we employed an X‐ray to observe the repair site at 2 weeks and 3 months post‐implantation. As illustrated in Figure 8b, immediately following implantation, the distinct porous structure of the scaffold was visible, with a clear boundary between the scaffold and the host bone. After 3 months, the boundaries began to blur, indicating the integration of the scaffold with the host tissue. Furthermore, the entire scaffold was gradually covered by new tissues, and the original porous structure was nearly imperceptible, suggesting the ingrowth of new bone within the material. Additionally, traces of fractures were observed in the HAP. During the experiment, we had six control subjects without implants. Four of these suffered fractures within 1‐month post‐surgery, and the remaining two fractured at ≈2 months post‐surgery with typical failure characteristics, as shown in the X‐ray images (Figure 8b). This further underscores the importance of material support in repairing segmental bone defects at weight‐bearing sites. Although ceramic materials cannot serve as primary load‐bearing structures, they facilitate the maintenance of overall stability. Particularly, during the repair process, the ingrowth of new tissue can further assist the scaffold in stabilizing the skeletal structure.

### 2.10 Morphology of New Bone Tissue

**Figure 9:**
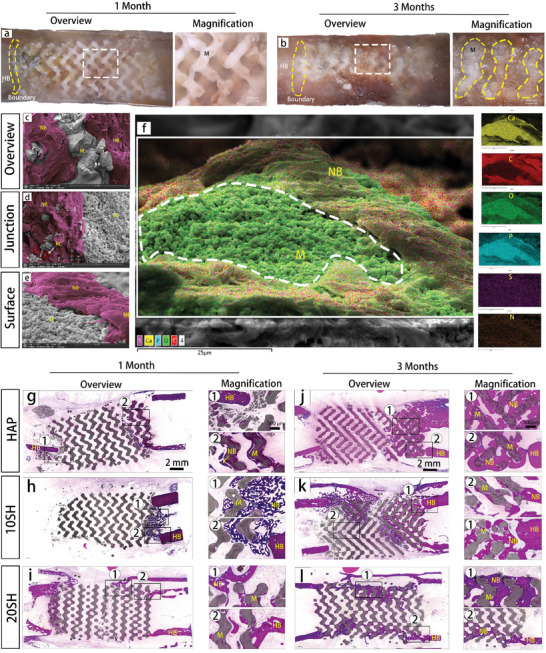
Morphology of new bone tissue. (a) Images of the scaffold and repair site after 1‐month post‐implantation, with the overall view on the left and a magnified view of the area framed in white on the right, where the light part represents the scaffold material. (b) Images of the scaffold and repair site after 3 months post‐implantation (HB: host bone, M: scaffold material, with the material‐tissue interface outlined by a yellow curvilinear frame). (c–e) Scanning electron microscopy (SEM) images of new bone tissue and the scaffold. Overall morphology is shown in (c), where new bone tissue and host bone are marked in red. The material‐tissue interface (d) and surface (e) were magnified for detailed observation. (f) Energy‐dispersive X‐ray spectroscopy (EDS) mapping of bone tissue adhered to the material surface, with color overlays representing Ca, C, P, O, S, and N. (g–i) Hematoxylin and Eosin (H&E) histological staining of 10 SH, 20 SH, and HAP after 1‐month implantation. The left column shows the overall H&E staining of the scaffold‐repaired femoral shaft truncation sites, with selected areas 1 and 2 magnified to observe the morphology of the new bone. (j–l) H&E histological staining after 3 months implantation. (HB: host bone, M: scaffold material, NB: new bone tissue).

Integration of the material and bone tissue was observed using optical microscopy. One month post‐implantation, the porous structure of the material was filled with newly formed tissue, and no fibrous encapsulation was detected (**Figure** **9**a). At this stage, the tissue was transparent with a low‐density, allowing clear visualization of the porous architecture of the material. A distinct demarcation between the left side of the scaffold and the host bone was observed. Three months after implantation, the material and bone tissue were tightly integrated, and the boundary between them was completely obscured. The color and morphology of the newly formed tissue within the scaffold closely resembled those of the host bone (Figure 9b). SEM and EDS were conducted to further investigate the interface between the material and the tissue. The new tissue contained not only carbon but also calcium and phosphorus. Combined with the histological sections, it can be preliminarily concluded that the newly formed tissue on the material surface was osseous (Figure 9f). As shown in Figure 9c–e, the new osteal tissue tightly enveloped the scaffold material, and the new tissue proliferated along the surface of the ceramic grains, substantiating the biocompatibility and bioactivity of the IWRCs.

One month after implantation, H&E staining was performed to evaluate the formation of new bone within the scaffolds. As shown in Figure 9g–i, evident new bone ingrowth with a depth of 3 mm was observed in HAP at the right end, where the lower right end of the scaffold and the bone tissue had fused. No new bone tissue was formed at the left end of the scaffold. A large amount of material debris was found next to the lower left cortical bone, suggesting that the fracture of the material was likely caused by the compression of the cortical bone. In contrast, new bone tissue was observed at the right end of 10 SH, achieving partial fusion with the host bone. In comparison, bone tissues grew from the outer edge of the scaffold toward the center in 20 SH, with a length of 6 mm. The newly formed bone tissue was dense and toward the host's cortical bone. At this time, the fusion with the host bone was distinct at the left end of the scaffold and noticeable at the right end. These results indicate that 1 month after implantation, the defect began to heal gradually, and the scaffold had partially fused with the host bone.

Three months after implantation, the growth of new bone in each group is shown in Figure 9j–l. Compared to 1 month, the new bone tissue in the scaffolds grew significantly, and the bone mass increased. In HAP, the new bone tissue was dense and concentrated at the left end of the scaffold, where it completely fused with the host cortical bone. However, no significant bone tissue was observed on the right side of the material, and the right end of the scaffold was not fused to the host. Additionally, on the lower‐right side of the scaffold, the cortical bone had a crushed layer with the scaffold's pore structure. Although the crushed material remained in close contact with the host cortical bone, no new bone ingrowth was observed. In 10 SH, new bone tissue grew significantly at both ends of the scaffold and spanned the entire scaffold, leaving only a 2–3 mm gap unhealed. Furthermore, the fusion of the scaffold with the host on the right side was more significant than that on the left side, where local fragmentation of the scaffold was observed without new bone tissue. In 20 SH, the scaffold structure was clear, and both ends were completely fused with the host bone. New bone tissue spanned almost the entire scaffold along the outer edge, and the direction of the tissue was close to that of the cortical bone of the host. The new bone tissue was dense and resembled the host's cortical bone, and a large number of Haversian canals were found within the new bone, indicating that the bone tissue matured.

Furthermore, in the immunohistochemical staining, the osteogenic‐related genes (Runx2, COL‐1α, BMP2, and OPN) and the angiogenic gene CD31 in the IWRC group had strong positive expression (Figures S14–S16, Supporting Information). In this study, experimental animals were intravenously injected with fluorescein and tetracycline hydrochloride 3 and 13 days before sample collection. The borders of the mineralized bone tissue were marked with green and orange fluorescence. A significant spacing between the two fluorescent lines indicated that the bone tissue growth was continuous (Figure S17, Supporting Information). The overall repair effect followed the order of 20SH > 10SH > HAP.

### 2.11 Mechanical Testing after Repair

**Figure 10:**
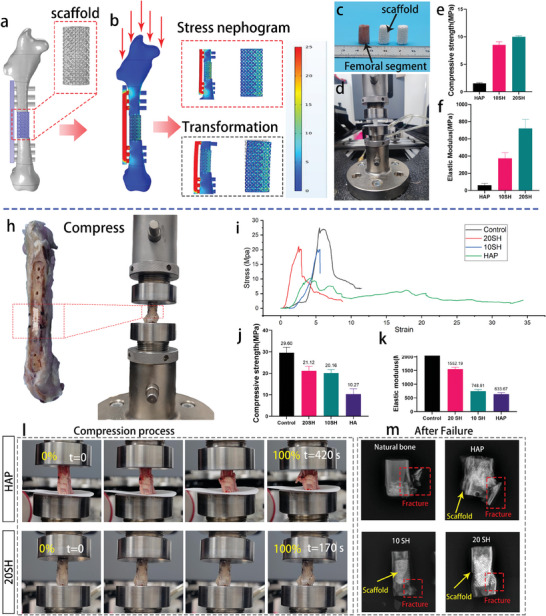
Biomechanical evaluation of bone repair at weight‐bearing sites. (a–c) Pre‐implantation finite element mechanical analysis of truncated bone animal models. A force of 150 N was applied to the femoral head for stress analysis of the repair system (a), with corresponding stress contour maps (b) and potential deformation patterns (c) calculated for the scaffold. (d–f) Compression tests of the personalized repair constructs for rabbit femur shafts, where the custom‐made scaffolds matched the external structure of the rabbit femur shafts (d), compression strength (e), and elastic modulus (f) were calculated, integrating the results with finite element simulations to predict the failure risk of the three scaffold groups. (h–l) Mechanical tests 3 months post‐implantation of 10 SH, 20 SH, and HAP, involving compression testing of femur shaft repair site sections containing the scaffolds (h), corresponding stress‐strain curves (i), and calculation of compressive strength (j) and elastic modulus (k), recording the scaffold failure process during compression (l) (0% indicates no deformation; 100% indicates complete failure; t denotes the time required for the sample to completely fail). (m) X‐ray images of natural bone tissue and the three scaffolds after failure (red frames mark the locations of bone fractures).

FEA was performed to assess the feasibility of the scaffold. The scaffold design model is shown in **Figure** **10**a, and supercritical bone defect repair scaffolds were 3D printed using HAP, 10 SH, and 20 SH according to the design. In the analysis, it was assumed that each rabbit weighed ≈3 kg, and when hopping, the legs bore a weight significantly heavier than their own. We set it to ten times the weight of the rabbit itself, that is, a total force of 300 N, with each side bearing 150 N. During the surgery, a steel plate was used for unilateral fixation (Figure 10a). A force of 150 N was applied to the top end of the rabbit femur to analyze the stress on the femur shaft, scaffold, and steel plate (Figure 10b). The results showed that the steel plate bore most of the force, with stress mainly distributed around the screw holes on the bone shaft, and the remaining pressure was borne by the scaffold, with the calculated compressive stress on it being 7.42 MPa. The stress on the scaffold was mainly distributed on the side opposite the steel plate, and the scaffold could fail from one side if further deformation was applied (Figure 10b). The compressive strengths of 10 SH and 20 SH scaffolds were 8.53 and 10.00 MPa, respectively, higher than the strength borne by the scaffold in the simulation experiment. Therefore, these materials can meet the usage requirements for large‐segment bone repair (Figure 10c–f). However, the compressive strength of pure HAP ceramics is only 1.5 MPa, which presents a significant risk of failure. This simulated result was in accordance with the experimental results.

The recovery status of bone mechanics can provide an intuitive evaluation of the bone repair process. Three months post‐implantation, we removed the samples and disassembled the internal fixation devices, such as the steel plate. Considering that the femur of the lower limb is the primary load‐bearing bone, we then cut the femoral shaft containing the material along the two screw holes in the middle and conducted compression tests. The bones implanted with steel plates were used as positive controls. The compression test layout and the morphology after failure are shown in Figure 10h. The compressed stress‐strain curve is shown in Figure 10i. For HAP, the scaffold material was compressed and deformed during the initial test stages. When the material was flattened, the bones at both ends came into contact, splitting the bone. It was evident from the photograph that the HAP scaffold was squeezed into a lump and protruded outside the bone. X‐ray images revealed that the HAP scaffold was completely compressed with a significant crack in the bone and a compressive strength of 10.27 MPa. In contrast, the bone with 20 SH scaffold first split at the bottom during compression and then expanded until complete failure. The X‐ray image indicated that after failure, the main body of the 20 SH scaffold remained intact, and its compressive strength was 21.12 MPa (twice that of the HAP ceramics after repair and close to 71.35% of the control bone). The HAP and 10 SH scaffolds, after 3 months of repair, could recover 34.7% and 68.11% of the mechanical support of the original bone, respectively. The compression processes of the control and 10 SH are shown in Figure S18 (Supporting Information).

## 3 Discussion

### 3.1 Mechanical Reinforcement of 3D‐Printed Porous Calcium Phosphate Ceramics

A porous structure is an indispensable factor for bioactive bone repair materials, which can provide suitable growth space for the tissue and ensure substance exchange.^[^ ^32^, ^33^ ^]^ Although porous CaP has an outstanding bioactivity, its poor mechanical properties significantly limit its clinical applications. Studies show that the optimum porosity to promote new bone growth is ≈60%–80%, and the ideal pore diameter is 200–800 µm.^[^ ^34^ ^]^ The sintering temperature of porous CaP ceramics is generally lower than 1200 °C to ensure a large number of micropores in the matrix.^[^ ^35^, ^36^ ^]^ These micropores, as stress concentration points, first crack under stress, causing the failure of the entire scaffold. Although the compressive strength of the dense HAP sintered at 1300 °C can reach 500 MPa, which is much higher than the cortical bone of the human body, such ceramics completely lose their bioactivity and have no application value in bone regeneration.^[^ ^37^ ^]^ In this study, a whisker‐reinforced structure was formed during the sintering process by regulating the in situ oriented growth of HAP grains. The simulation results show that the in situ grown whiskers can effectively slow down the stress concentration at the interface of the microporous material, which effectively alleviates the negative impact of the microporous structure on the mechanical properties. Moreover, CaSO_4_ promotes the fusion between the HAP grains, and the strength at grain boundaries is significantly enhanced. Therefore, the fracture modes of the IWRC include trans‐granular and intergranular fractures, which can consume more fracture energy.

Compared with the reinforcement by adding external whiskers, the whisker structure formed by in situ oriented growth is more closely combined with the matrix with lower internal stress. The existing 3D printing technology, especially SLA/DLP, is not suitable for HAP whiskers because the addition of pre‐prepared whiskers in the bio‐ink severely reduces fluidity and printing accuracy.^[^ ^38^, ^39^, ^40^, ^41^ ^]^ In our study, CaSO_4_ was added to the slurry as an inducer to promote whisker growth, which did not change the fluidity and ensured the normal progress of printing. Subsequently, a rod‐like whisker structure was formed during the sintering process through the oriented growth of HAP grains. The growth of whiskers depended on the content of CaSO_4_. When the content was less than 5%, whisker growth was not evident. In this study, although the regulatory mechanisms of in situ preferential grain growth and their effects on the mechanical enhancement of hydroxyapatite ceramics were explored, the investigation into the mechanisms underlying grain orientation growth was lacking. In future research, we aim to explore the kinetic factors influencing the oriented growth of hydroxyapatite ceramics regulated by calcium sulfate. Molecular dynamics simulations will be used to examine the interactions between different crystal facets of CaSO_4_ and HAP, thereby studying the effects of CaSO_4_ on the growth characteristics of HAP crystals on various planes.

### 3.2 IWRC Enhance Osteogenesis

Porous structure and chemical composition are two fundamental factors that affect bioactivity.^[^ ^42^ ^]^ Porous scaffolds, which serve as bridges for tissue growth, should possess multilevel pore structures to provide suitable habitats for key factors involved in tissue growth, such as proteins, cells, and blood vessels.^[^ ^43^ ^]^ We used 3D printing technology to prepare scaffolds with customized macroporous structures. On this basis, by growing whiskers at a lower temperature (900 °C) and maintaining the sintering temperature at 1150 °C, we retained the capillary micropores on the scaffold surface.

CaP is advantageous chemically because of its ability to release active ions.^[^ ^44^ ^]^ HAP, as the thermodynamically stable phase of CaP, has a low hydrolysis rate (≈6.62 × 10^−126^) and degrades very slowly in the body. BCP ceramics, owing to the inclusion of fast‐degrading TCP, degrade slightly faster than HAP and release more calcium and phosphate ions, exhibiting higher biological activity than HAP.^[^ ^45^, ^46^ ^]^ Ca^2+^ is not only an important component of the inorganic part of the skeleton but also a signaling factor in living organisms that can regulate the transcription of genes. Ca^2+^ plays an important role in cell proliferation and differentiation. Typically, the concentration of Ca^2+^ in cells is ≈100 nm, which increases to 500–1000 nm upon activation. An increase in Ca^2+^ concentration in the extracellular environment can increase the intracellular Ca^2+^ concentration through L‐type and non‐L‐type calcium channels and Ca^2+^ receptors, thereby activating osteogenesis‐related signaling pathways.^[^ ^47^ ^]^ In this study, owing to the introduction of CaSO_4_, the release rate of Ca^2+^ was significantly increased. In an in vitro experiment in the absence of osteogenic inducers, IWRC upregulated the expression of genes related to calcium ion binding and promoted osteogenic differentiation. In addition, within the back muscles of Beagle dogs, IWRC significantly promoted the expression of calcium ion‐regulated proteins (F2 and FFAR2) and ossification‐related proteins (AHSG, ACP5, IGF2, SPP1, and MGP), demonstrating improved osteoinductivity and bioactivity. Consequently, in the repair of supercritical bone defects, the regenerative ability of IWRC was significantly enhanced, and new bone tissue penetrated the entire scaffold in just 3 months to repair the defect.

## 4 Conclusion 

In this study, high‐precision porous ceramic scaffolds were printed using a CaSO_4_/HAP ceramic slurry, followed by controlling grain orientation growth during subsequent sintering to achieve a mechanically reinforced grain arrangement structure. The study investigated the mechanisms by which calcium sulfate regulates the in situ oriented growth of HAP grains and their associated mechanical enhancement effects. It was found that CaSO_4_ initially promoted grain merging within the ceramic, strengthening the grain boundary binding strength. Moreover, the in situ whiskers effectively reduced stress concentration within the porous material, resulting in a 5–10 times increase in the compressive strength of the porous CaP ceramics. Additionally, CaSO_4_ was observed to enhance the release of active Ca^2+^ in IWRC ceramics, significantly improving the osteoinductivity of HAP. Transcriptomic and proteomic results indicated that IWRC could activate the calcium ion pathway and the expression of osteogenesis‐related proteins. In preclinical animal experiments, IWRC achieved the repair of supercritical bone defects within just 3 months, with mechanical recovery exceeding 70% of autologous bone. This study reconciled the contradiction between the bioactivity and mechanical strength of porous ceramics, successfully achieving the dual optimization of the bioactivity and mechanical strength of CaP ceramics.

## 5 Experimental Section

### Scaffold Preparation

First, 50 g of polyurethane acrylate, 15 g of polyethylene glycol diacrylate, and 1 g of BYK‐2155 were thoroughly mixed. Subsequently, 60% HAp was added to the aforementioned mixture in a planetary ball mill, and the mixture was milled at a speed of 240 rpm for 15 h. Next, 10, 20, and 30% of calcium sulfate dihydrate (CaSO_4_·2H_2_O) based on the HAP mass was added, and milling was continued for 2 h. Two grams of TPO was then added, and milling was continued for an additional 2 h. The slurry and grinding balls were separated, and the debubbling process was carried out using a vacuum‐defoaming device to obtain the printing slurry.

After printing, the samples were subjected to ultrasonic cleaning with anhydrous ethanol to remove uncured slurry, with precautions taken to avoid contact with water during the cleaning process. The samples were then placed in a muffle furnace for degreasing and sintering, following a temperature profile: ramping at 5 °C min^−1^ to 300 °C and holding for 6 h, further ramping at 5 °C min^−1^ to 600 °C and holding for 2 h, subsequent ramping at 5 °C min^−1^ to 900 °C and holding for 10 h, and finally ramping at 5 °C min^−1^ to 1150 °C and holding for 2 h, followed by natural cooling to room temperature.

### Material Microstructure

The microstructure of the samples was observed and photographed via scanning electron microscopy (SEM; JSE‐5900LV, Japan). All ceramic samples were sputter‐coated with gold for 70–140 s before the analysis. The composition of the samples was analyzed using the EDS extension function of the scanning electron microscope.

### Ion Release

Porous materials of the same macro size were placed in a 0.1 m Tris‐HCl buffer solution to create a system that was 30 times their own weight. This solution was then oscillated at 37 °C for 1, 2, 4, 8, and 16 days. Next, the supernatant was extracted and analyzed via inductively coupled plasma–optical emission spectrometry (ICP‐OES; 5100 SVDV, Agilent, USA).

### In Vitro Degradation

The in vitro degradation rate of the scaffold was tested by using a 0.1 m Tris‐HCl buffer as the medium. The tested sample was a porous circular sheet with a diameter of 2 mm and a diameter of 8 mm, and the material porosity was 68%. The porous sample was placed in a 50 mL centrifuge tube, Tris‐HCl buffer was added at a ratio of 1 g/30 mL, and the tube was placed in a 37 °C shaker for 1, 4, 7, and 14 days. During this test, the buffer solution was replaced once every 2 days. At each test point, the sample was removed, washed with water three times, placed in an oven until a constant weight was achieved, and the material mass difference was calculated.

### Mechanical Simulation

The mechanical simulation was carried out using the mechanical simulation section of COMSOL Multiphysics (version 5.6). Because the investigated material was novel and does not have standard material parameters, the Young's modulus of the material was uniformly set to 281 GPa and the Poisson's ratio was set to 0.25 in the simulation experiment. A 2D single‐layer simplified model was used for convenience of analysis. According to the SEM results, the inside of the model mainly consisted of pores, equiaxed particles, and columnar whiskers. The raw HA particles were simplified as spherical and the columnar whiskers were simplified as cylindrical systems. Considering that the model did not undergo plastic deformation, it was a linear elastic model.

### Nanomechanics

The surface nanomechanics of the materials were measured using atomic force microscopy (AFM; MFP‐3D‐BIO, Asylum Research, USA). The force‐displacement curves in the grains and at the grain boundaries of the ceramics were measured to calculate the Young's modulus.

### XRD

The phase structures of the raw materials and ceramic scaffolds were analyzed using X‐ray diffraction (XRD; Philips X'Pert 1 X‐ray diffractometer, Netherlands) after grinding the ceramic scaffolds into powder. Using a Cu target with a voltage of 30 kV and current of 20 mA, the samples were scanned at a rate of 0.05 °/s in the range of 10°–70°.

### Compression Test

The compressive strength of the ceramic samples was analyzed using a universal testing machine according to the standard ISO13314:2011 (E). The test samples were cylinders with dimensions of Φ6.5 × 13 mm that were compressed at a rate of 1 mm min^−1^ along the axis of the cylinder until failure. Each group consisted of at least three parallel samples. Stress–strain curves were calculated based on the load and displacement during the compression process.

### In Vitro Proliferation and Differentiation

All cell extraction, media change, passaging, and planting operations in the experiment were performed on a sterile workbench. All cells were cultured in an incubator at 37 °C with 5% CO_2_, and the media was changed or passaged every other day. A density of 5 × 10^4^ BMSCs cells was seeded on the surface of the material. After incubation for 15 min, 1 mL of α‐MEM complete medium per well was used for routine culturing. At 1, 3, and 7 days, the CCK‐8 reagent kit was used to detect the cytotoxicity of the material and determine the proliferation, and each group of materials had three parallel samples. Fluorescein diacetate (FDA)/propidium iodide (PI) was used to fluorescently stain the cells, and the morphology of the live/dead cells was observed by confocal laser scanning microscopy. Similarly, a density of 5 × 10^4^ BMSCs cells was seeded on the surface of the material for the osteogenic differentiation test. When the cells at the bottom of the well plate reached 80% confluence, the medium was replaced with an osteogenic induction medium, and the culturing was continued for 10 days to measure the alkaline phosphatase (ALP) activity using an ALP activity kit (Beyotime Co., China). On the 14th day, the calcification nodules of the cells were observed using alizarin red S (ARS) staining. Further, immunofluorescence staining was employed to label the expressions of Runx2, Collagen1 (Col1), Osteocalcin (OCN), and Osteobridging protein (OPN).

### Transcriptomic Analysis

The co‐culturing of BMSCs and the scaffold was carried out using a six‐hole Transwell culture plate, in which the 20 SH and HAP porous scaffolds (*n* = 3) were placed in the Transwell chamber, and 5 × 10^4^ BMSCs were seeded at the bottom of the well plate. The ordinary culture medium α‐MEM without any induction components was used for the routine cell culture. After 21 days, the cells were lysed using trizol and RNA was extracted for transcriptomic analysis.

### Proteomics Analysis

20 SH and HAP porous ceramics were implanted into the back muscle of beagle dogs, and the samples were removed after 1 month for 4D‐DIA proteomic analysis, with three samples in each group. The extra tissue outside the scaffold was removed, the frozen samples were added to liquid nitrogen, fully ground, and the protein was extracted. After protease digestion, liquid chromatography‐mass spectrometry was used to perform mass spectrometry analysis on the peptide segments. The proteins corresponding to the peptide segments were quantified in relation to one another by comparing the signal intensity of the corresponding peptide segments in different samples.

### Ectopic Osteoinduction

The back muscle model for beagle dogs was used to verify the osteoinductive properties of the investigated material. This study was approved by the Animal Ethics Committee of Sichuan University (Ethics registration number: 20230310045). The beagle dog (male, 8 kg) was purchased from Chengdu Dasuo Experimental Animal Co., Ltd., and raised in the Experimental Animal Center of the West China Science and Technology Park of Sichuan University. Under fasting conditions, the animal was fully anesthetized using an intraperitoneal injection of a 3% pentobarbital sodium solution. After disinfection and sterilization, the back skin and muscle were exposed, a small hole ≈2 cm was cut, and the four sets of sterilized brackets were placed inside. The sample diameter was 6 mm, the height was 10 mm, and the porosity was 68%. Each group had at least three samples. After the operation, the experimental dogs were raised until 3 months later, and then euthanized. The implanted samples were removed for photography, omics staining, and the detection of osteogenic gene expression.

### Establishment of a Large Bone Defect Animal Model

This experiment was conducted under the approval and guidance of the Animal Care and Use Committee of Sichuan University (Ethics registration number: 20230310045). Forty‐eight rabbits were randomly divided into four groups: a blank control group and three material groups (HAP, 10 SH, and 20 SH), with six rabbits in each group. After successful anesthesia, the surgical area was prepared by shaving the skin 3 cm below the knee joint and 4 cm below the greater trochanter of the femur. A curved incision ≈4–5 cm in length was made along the lateral side of the knee joint, taking care to protect the lateral collateral ligament of the knee joint and the joint capsule. A cutting point 15 mm from the middle of the bone was marked, and the bone was cut using a wire saw at the marked position. The material was placed and tied behind the steel plate using surgical silk, and the bone and steel plate were then reset. Screws with dimensions of Φ 2.4 mm (L14), Φ 2.4 mm (L16), and Φ 2.4 mm (L18) were sequentially used to fix the bone and the steel plate from the middle to the sides of the steel plate. The surgical incision was sutured layer‐by‐layer after rinsing with a 0.9% saline and iodine tincture, and the skin was disinfected. After 1 and 3 months of implantation, the experimental animals were euthanized, and the samples were removed for photography, histological staining, and biomechanical analysis.

### Compression Testing of the Femur

After 3 months of implantation, specimens from the healthy femur, HAP, 10 SH, and 20 SH groups of rabbits were obtained. The proliferative tissue around the steel plate was removed, and the screws and internal fixation steel plate were removed. The experimental sample was extracted along the positions of the two central screws, and the remaining bone shaft was cut to the same length as the experimental samples and reserved as a control sample. Another segment of the healthy femur from the opposite side of the experimental rabbits was taken as a positive control group, cut to the same length as the experimental samples. Compression testing was conducted using a universal mechanical testing machine with a loading speed of 1 mm min^−1^. The test was terminated when the stress value sharply decreased or when the sample exhibited obvious fracture.

### Statistical Analysis

All data in this section were analyzed using GraphPad Version 8.0 (GraphPad Prism). Two‐way analysis of variance was employed to assess the statistical significance of differences among the groups, with ^*^ *p* < 0.05, ^**^ *p* < 0.01, ^***^ *p* < 0.001, and ^****^ *p* < 0.0001 indicating statistically significant differences.

## Conflict of Interest

The authors declare no conflict of interest.

## Ethics Approval Statement

This animal experiment was conducted under the approval and guidance of the Animal Care and Use Committee of Sichuan University (Ethics registration number: 20230310045).

## Supporting information



Supporting Information

Supplemental Movie 1

## Data Availability

The data that support the findings of this study are available from the corresponding author upon reasonable request.
