# Supplementary material for: 3D‐Printed Bioceramic Scaffolds Reinforced by the In Situ Oriented Growth of Grains for Supercritical Bone Defect Reconstruction
Source: Adv Sci (Weinh). 2024 Nov 13;12(2):2408459. doi: 10.1002/advs.202408459 (PMC11727126; doi:10.1002/advs.202408459)
Supplement: Supplementary file 1 — Supporting Information [file ADVS-12-2408459-s002.docx]

**3D-Printed Bioceramic Scaffolds Reinforced by the In Situ Oriented Growth of Grains for Supercritical Bone Defect Reconstruction**

*Corresponding author. Email: Changchun Zhou: [changchunzhou@scu.edu.cn;](mailto:changchunzhou@scu.edu.cn;%20Yujiang) Yujiang Fan: [fan_yujiang@scu.edu.cn](mailto:fan_yujiang@scu.edu.cn)

**This file includes:**

Supplementary Text

Fig. S1 to S18

Movies S1

Supplementary Text

**Materials and Methods**

**Characterization of IWRC**

The microstructure of the samples was observed and photographed using a scanning electron microscope (SEM, JSE-5900LV, Japan). All ceramic samples were sputter-coated with gold for 70-140 seconds before the analysis. The composition of the samples was analyzed using the EDS extension function of the scanning electron microscope (SEM, JSE-5900LV, Japan). The surface morphology and the surface height difference were measured by atomic force microscope (AFM, Dimension ICON, Germany).

**ALP Colorimetric Detection.**

The BMSCs cells of 5🞨10^4^ were inoculated on the surface of the scaffold. Firstly, the complete α-MEM medium was used for routine culture. When the cells at the bottom of the well plate fused by 80%, the Osteoinduction medium was replaced and cultured until the 10th day. The upper layer of the medium was discarded, and the scaffold was washed three times with PBS to remove dead cells. After adding 1 ml of 4% paraformaldehyde solution for fixation for 10 minutes, the scaffold was washed three times with PBS. 300 μL of ALPchromogenic working solution was added to each well and incubated for 60 minutes. The chromogenic working solution was aspirated, the scaffold was washed three times with PBS to terminate the reaction. The scaffold was observed and photographed using a stereo microscope.

**Calcein and Tetracycline Hydrochloride Fluorescent Labeling.**

During the process of new bone formation, there will be deposition of calcium salt minerals. Calcein and tetracycline hydrochloride can chelate with calcium ions in the body and deposit together at the edge of the new bone. Working solutions of calcein (6 mg/kg) and tetracycline hydrochloride (30 mg/kg) were intravenously injected respectively 3 days and 13 days before the experimental animals were sacrificed. After sacrifice and sampling, hard tissue sections with a thickness of approximately 150 μm were obtained, and observed and photographed under the excitation light under a laser confocal microscope.

**Cell Adhesion Testing**

1×10^4 bone marrow-derived mesenchymal stem cells (BMSCs) were seeded on the surfaces of the four scaffold groups and cultured for 24 hours before observation using a scanning electron microscope. Prior to measurement, the cells adhered to the scaffolds needed to be fixed and dehydrated. One milliliter of paraformaldehyde solution was added to each well and fixed at room temperature for 5 minutes. Subsequently, dehydration was performed using a gradient of ethanol (40%, 60%, 75%, 90%, 100%). Finally, supercritical carbon dioxide drying was employed to remove any residual moisture from the scaffolds and cells. Prior to observation using the scanning electron microscope, a gold sputtering treatment was performed to enhance the conductivity of the scaffolds.


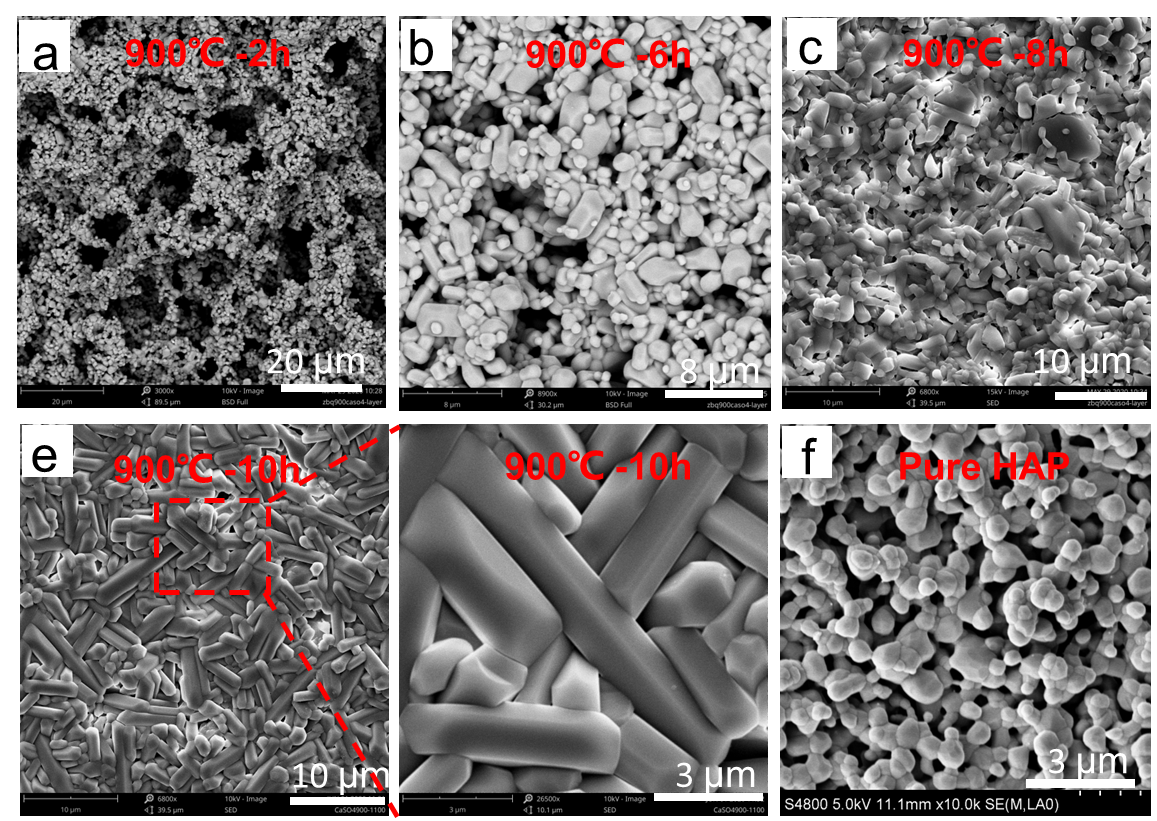


Fig. S1. The regulation of in-situ whisker growth in 50SH. (a-e) The 3D printed 50 SH ceramic green body after debinding, was sintered at 900°C for 2 h, 6 h, 8 h, and 10 h, respectively, and then at 1150 °C for 2 h. (f) The HAP was sintered at 900°C for 10 h, and then at 1150 °C for 2 h.


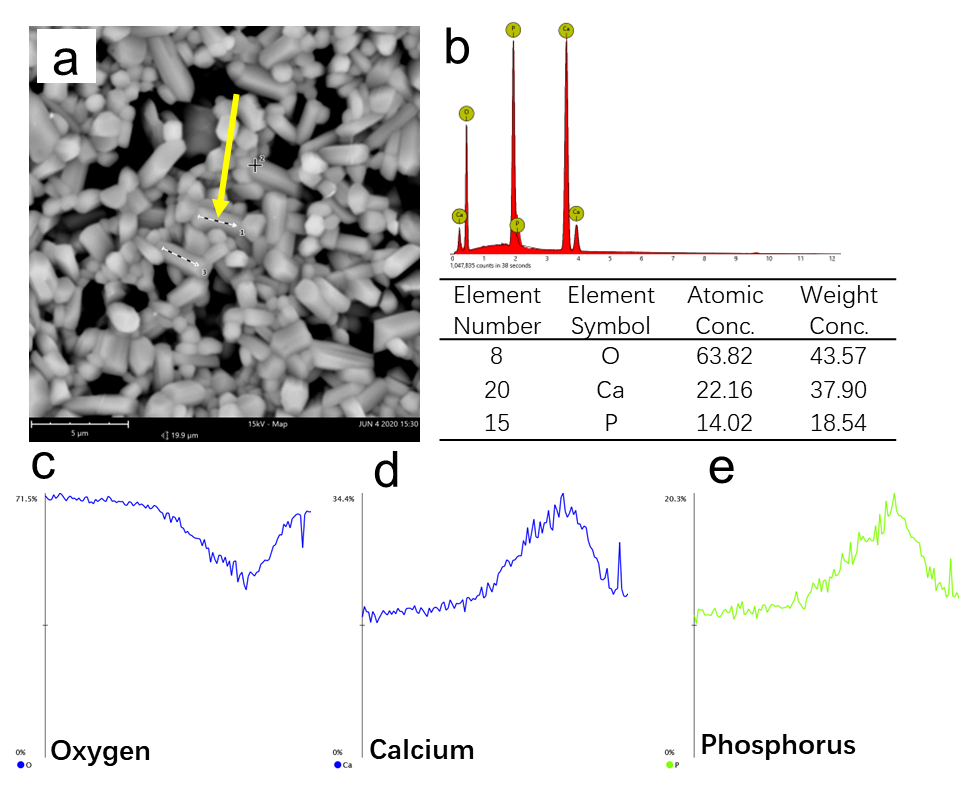


Fig. S2. Elemental analysis of in situ whiskers. (a) The morphology and location of the EDS line scanning. (b-e) The element peak diagram of in situ whiskers.


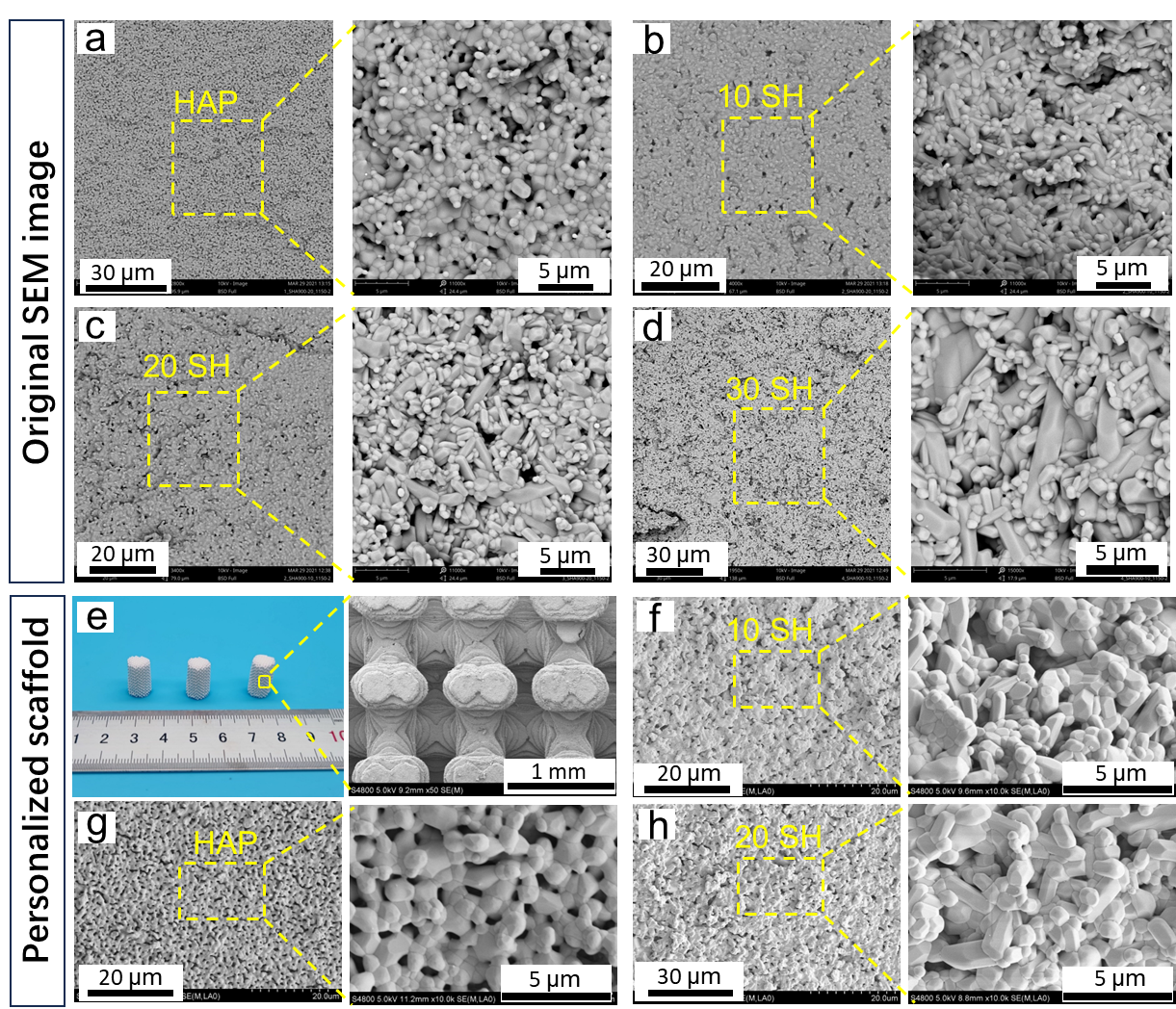


Fig. S3. Fabrication of IWRC. (a-d) Microstructural morphologies of four groups of materials: HAP, 10 SH, 20 SH, and 30 SH. (e-h) Microstructural morphologies of personalized porous scaffold for supercritical bone defect regeneration: HAP, 10 SH, 20 SH.


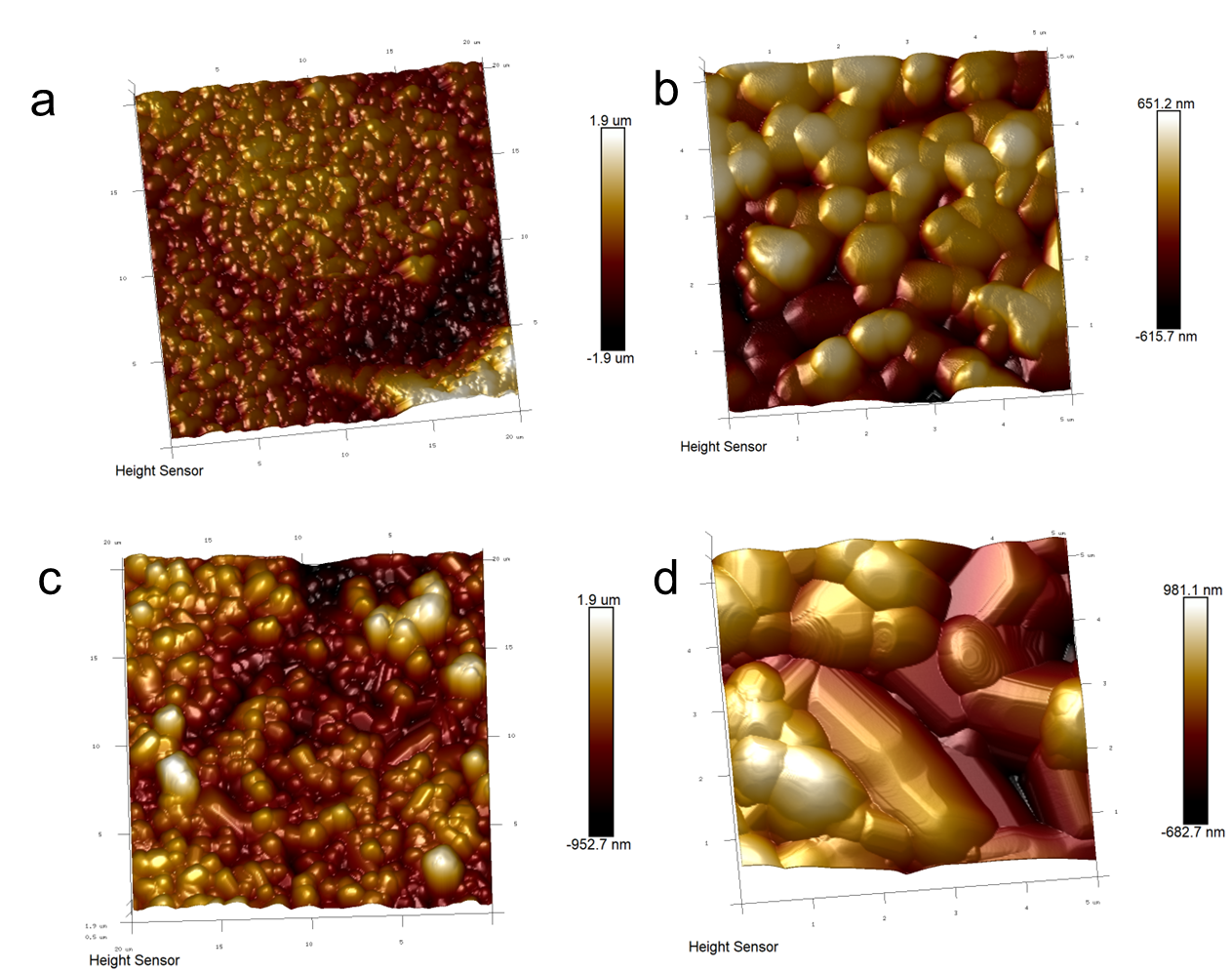


Fig. S4. The 3D graph of the surface height of IWRC. (a-b) HAP (c-d) 20SH


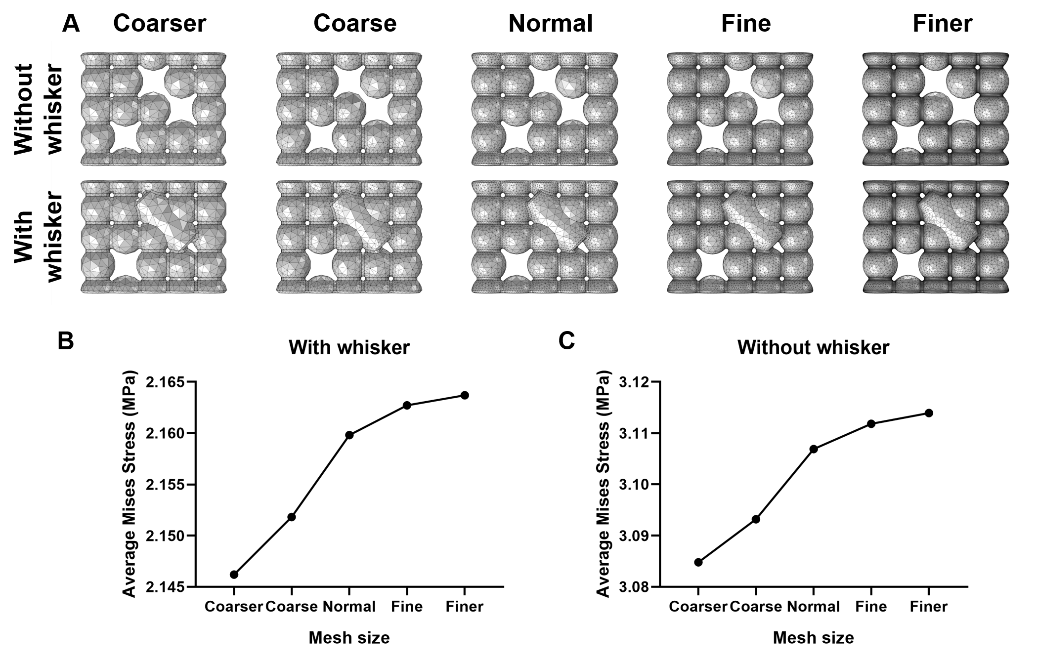


**Fig. S5. The mesh sensitivity analysis.**


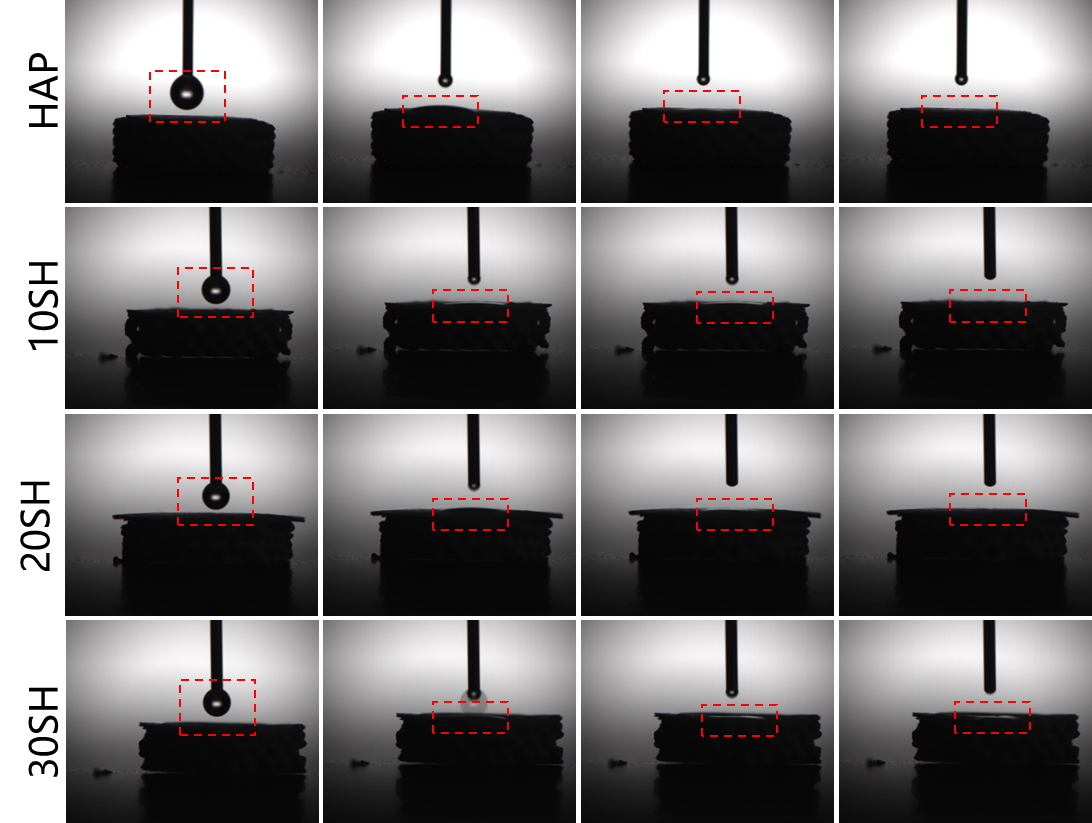


**Fig. S6. Water contact angle testing.** The testing process of the water contact angle is shown from left to right. It can be observed that once the water droplet contacts the material surface, it is quickly absorbed. This is due to the presence of numerous micropores on the material's surface, combined with its good hydrophilicity, which allows the droplet to be rapidly absorbed under capillary action.


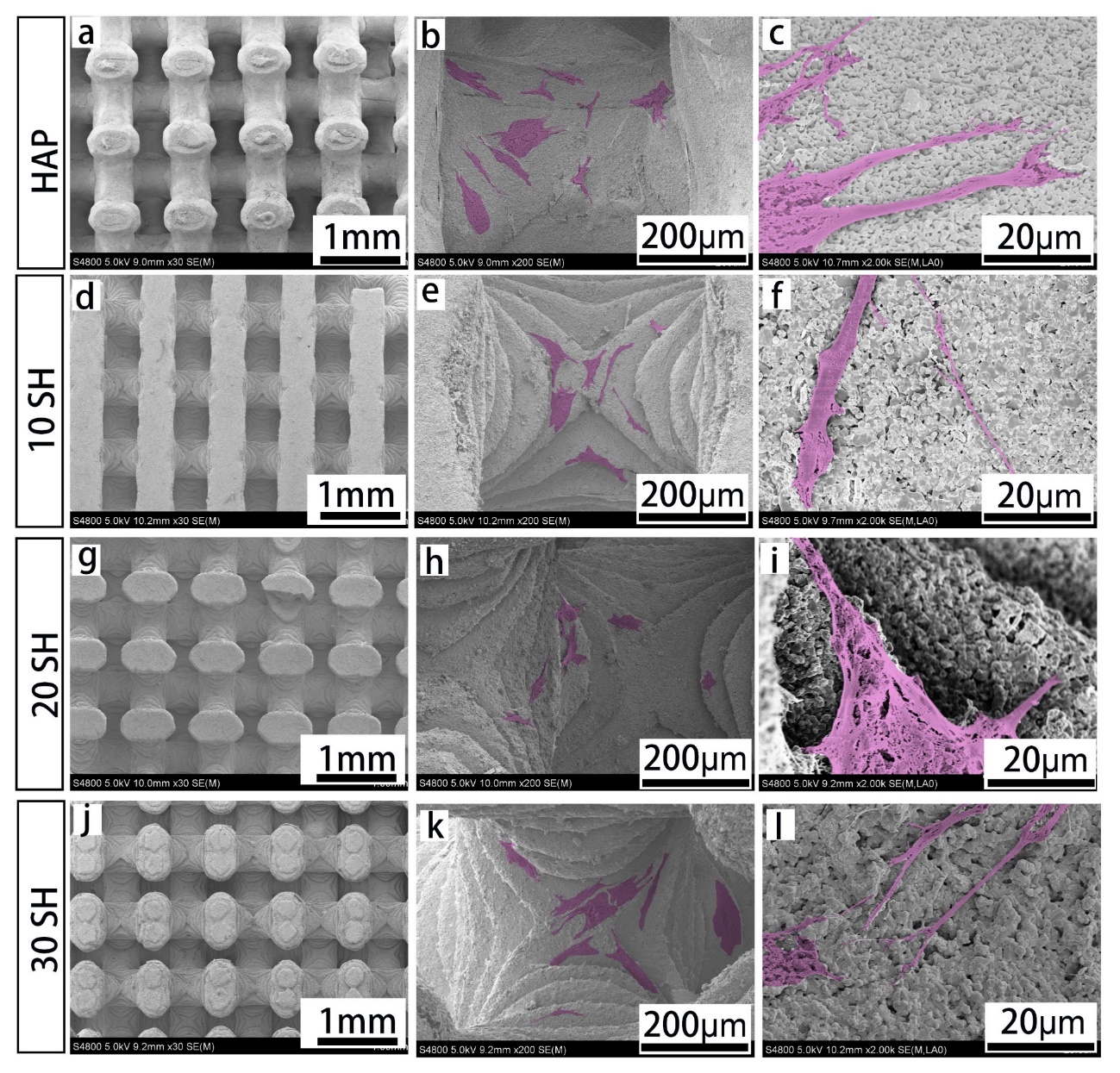


Fig. S7. Cell adhesion testing. (a-c) HAP. (d-f) 10SH. (g-i) 20SH. (j-l) 30SH


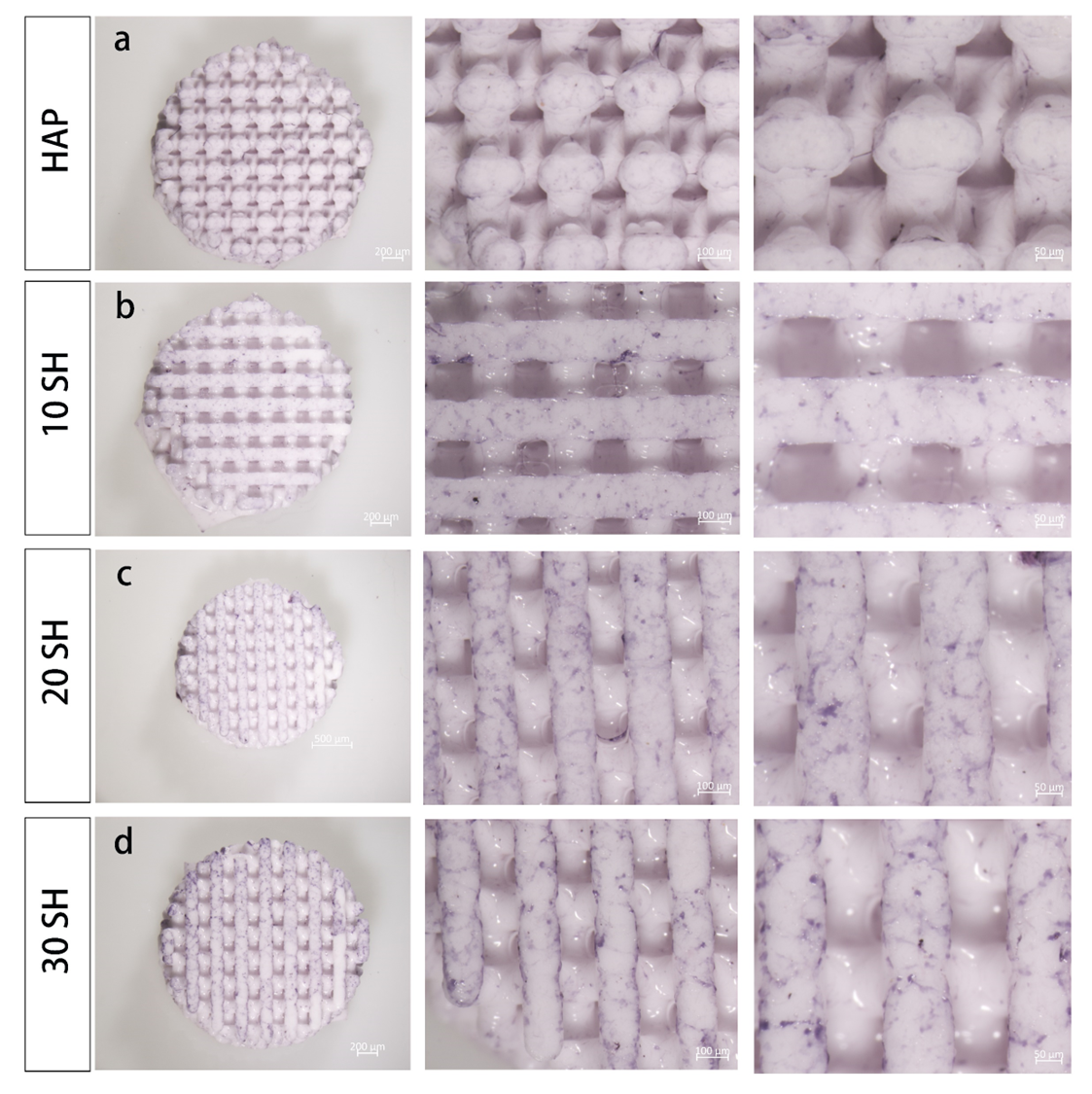


Fig. S8. The qualitative detection of ALP for the in vitro osteogenic differentiation ability of IWRC


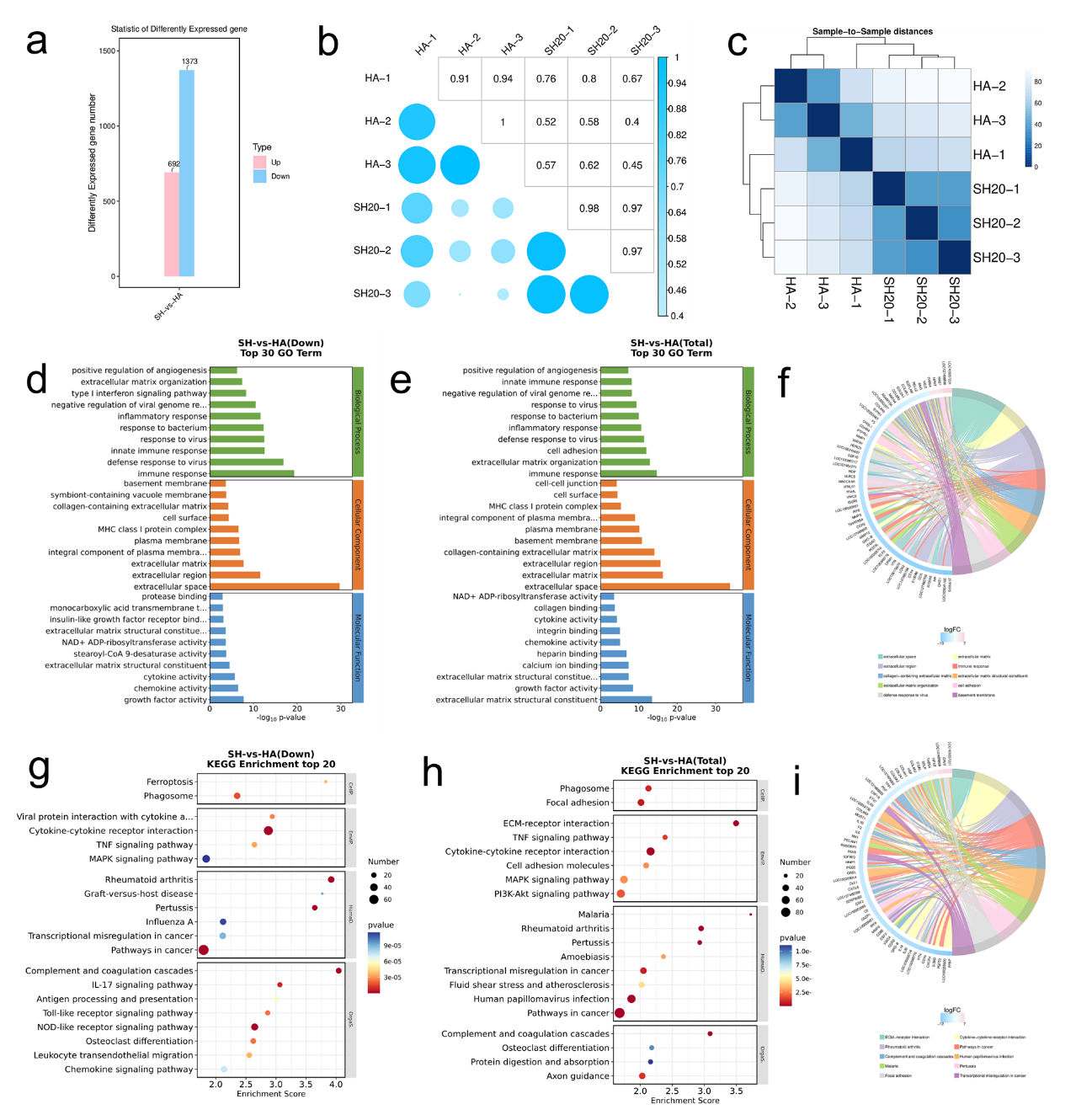


Fig. S9. Transcriptomic analysis for BMSCs osteogenic differentiation. (a) The statistical column chart of differentially expressed genes in 20SH and HAP. (b) The heat map of correlation coefficients among samples. (c) The result of Sample-to-Sample clustering analysis. (d-e) The result of GO enrichment analysis. (g-i) The result of KEGG enrichment.


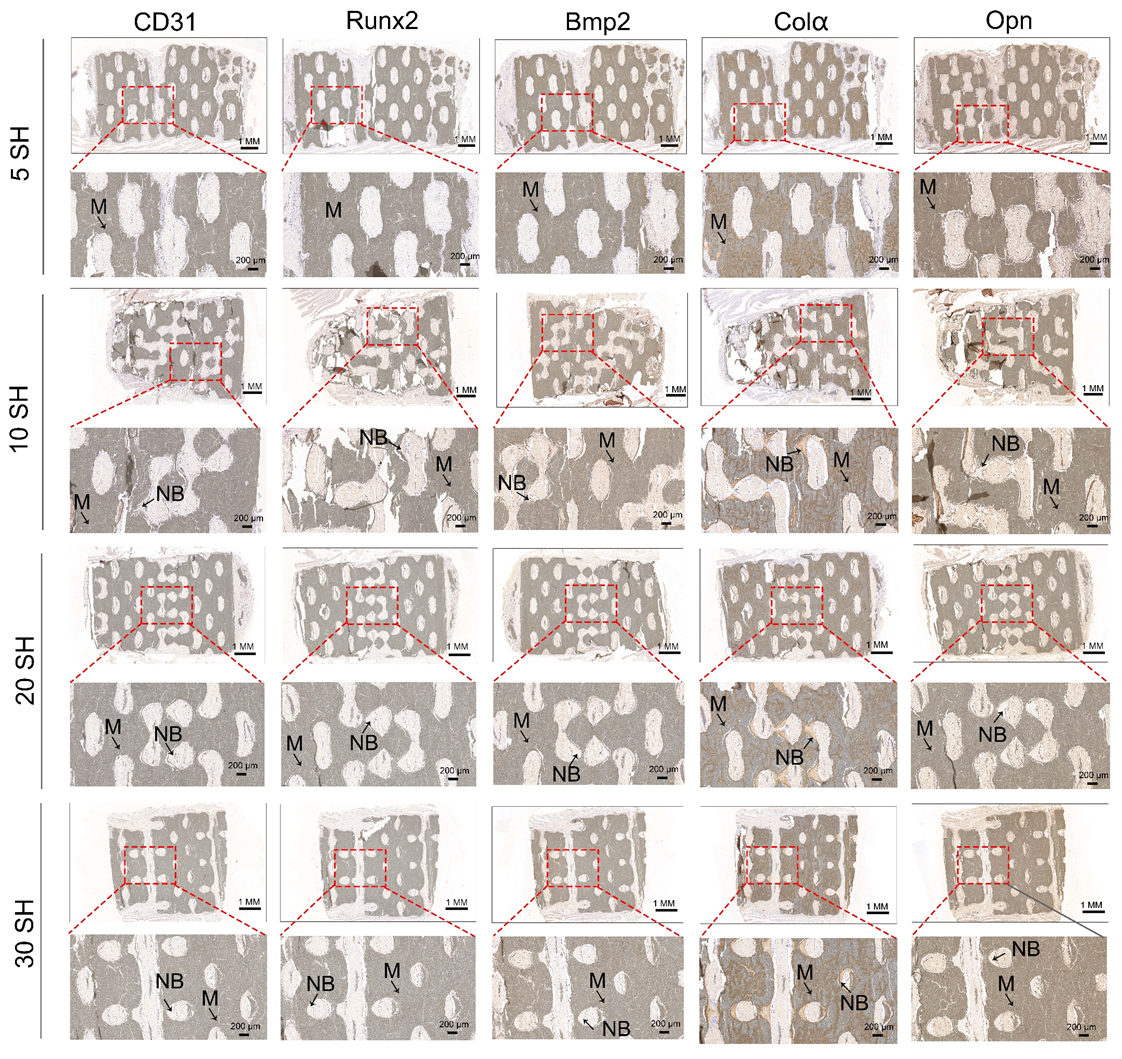


Fig. S10. Immunohistochemical staining of heterotopic osteogenesis. Mark the osteogenesis-related protein as CD31, Runx2, Bmp2, Colα, and Opn, among which the positive expression is brown (M: materials, NB: new bone).


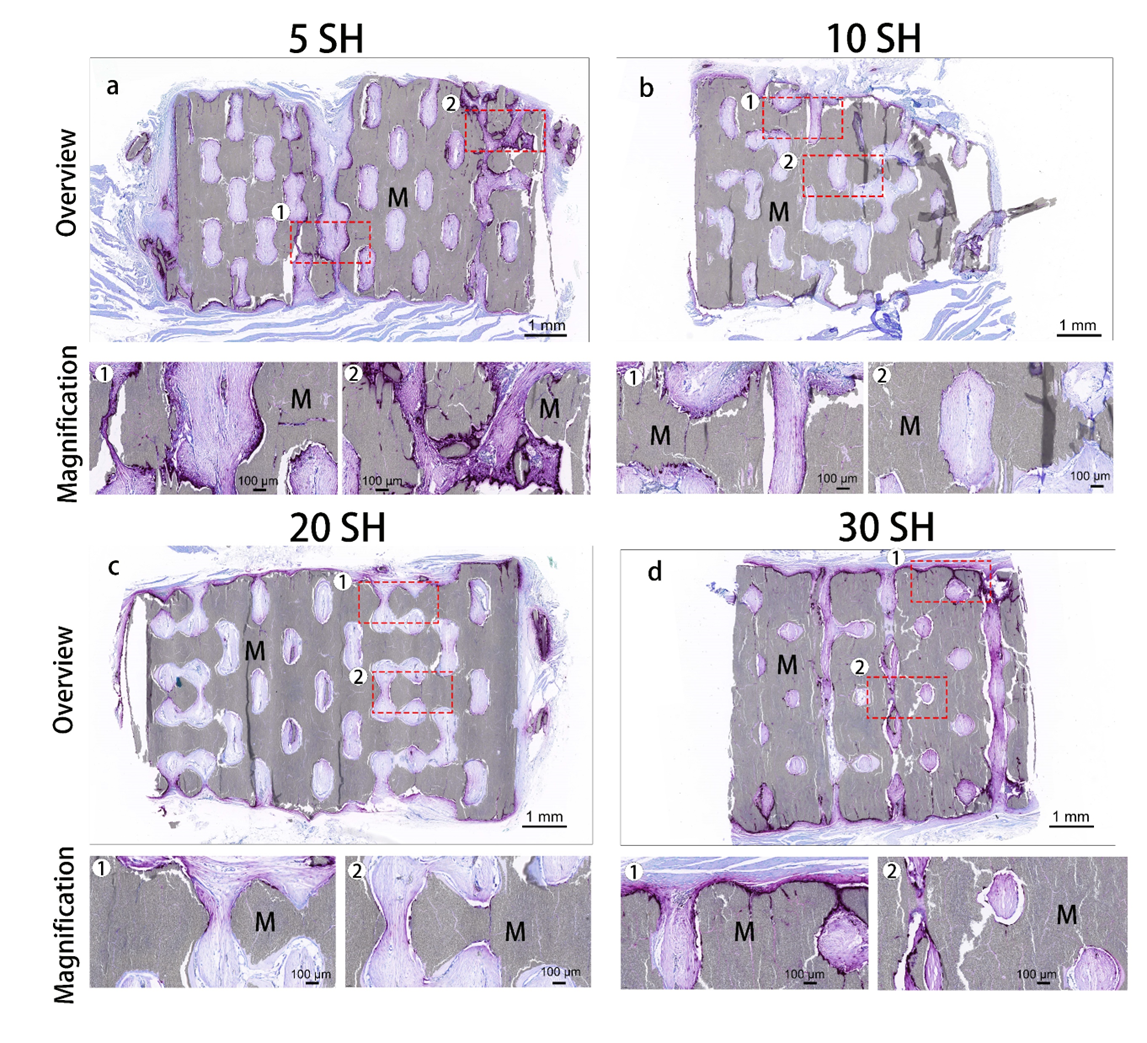


Fig. S11. The TRAP staining of heterotopic osteogenesis. The positive expression is red (M:materials).


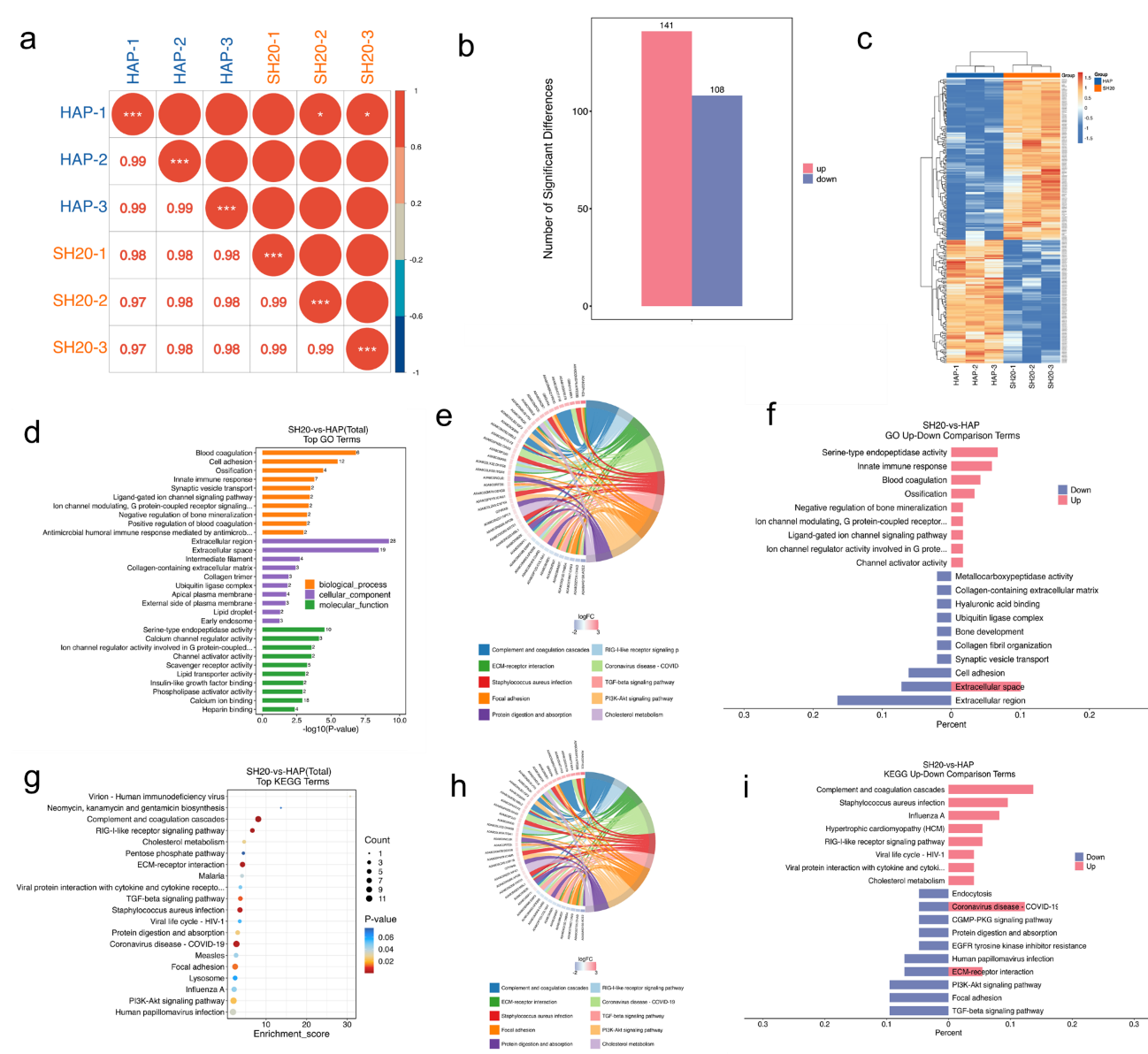


Fig. S12. Proteomic quantitative analysis of osteoinductivity in muscle tissue. (a) The correlation between different samples in HAP and 20SH.(b) Graph of differential protein statistics.(c) Cluster heat map of differential protein expression (sample clustering).(d) Analysis of bar chart for GO enrichment.(e) Chord diagram for GO enrichment analysis.(f) Comparison diagram of up-and-down regulated entries in GO enrichment analysis.(g) Bubble chart of top 20 in KEGG enrichment analysis.(h) Chord diagram for KEGG enrichment analysis.(i) Comparison diagram of up-and-down regulated entries in KEGG enrichment analysis.


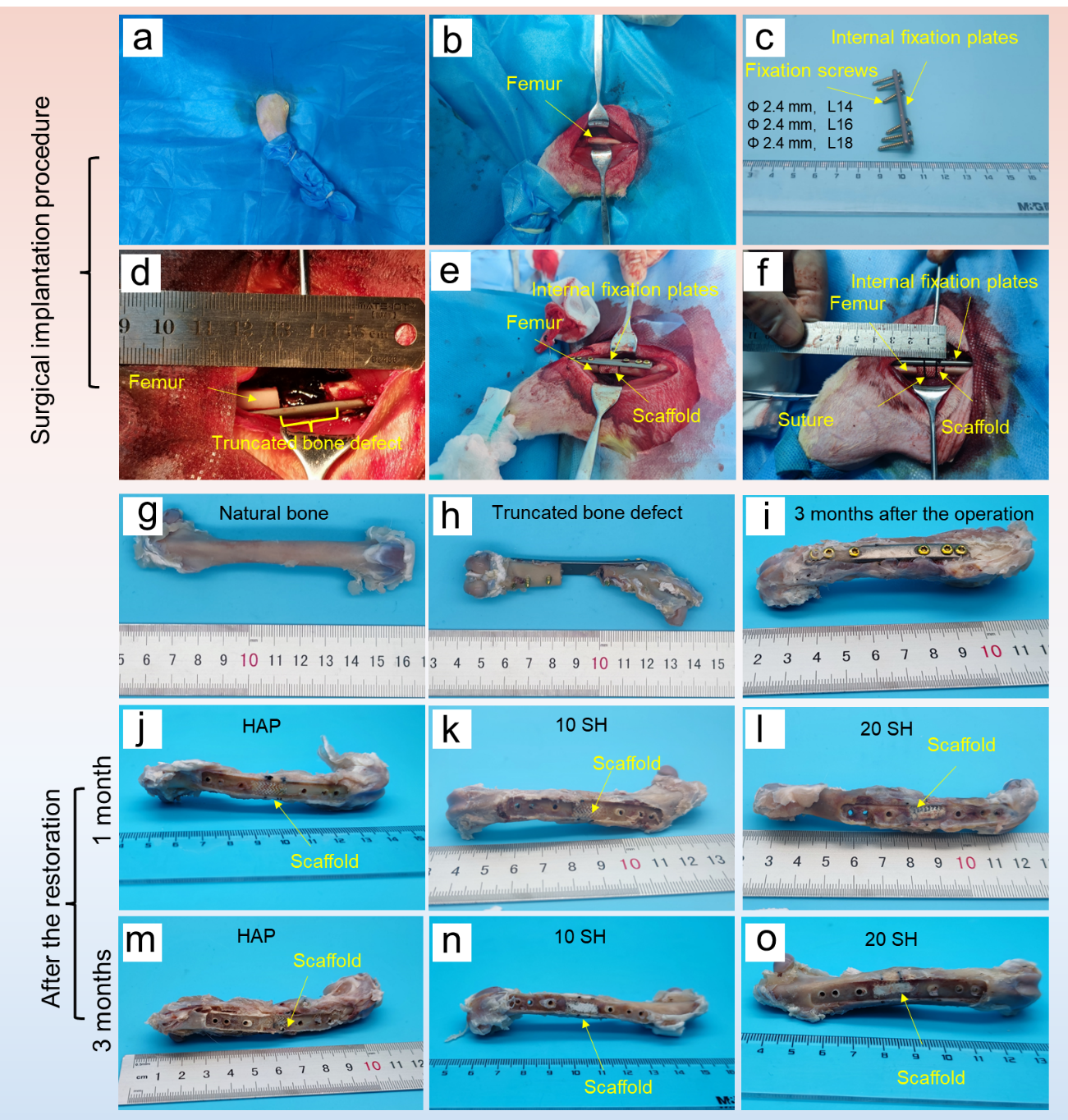


Fig. S13. The establishment of the supercritical bone defect model and the samples after 1, 3 months of implantation. (a) Preparation of the skin, disinfection. (b) Opening, exposing the femoral shaft. (c) Inside fixation system (d) Establishment of the defect model. (e-f) Implantation of the scaffold. (g) Healthy rabbit femur. (h) Supercritical truncated bone defect model. (i) The condition after the scaffold repair for three months without disassembling the internal fixed steel plate. (j-o) The appearances of HAP, 10SH, and 20SH separately implanted into the defect for 1 and 3 months.


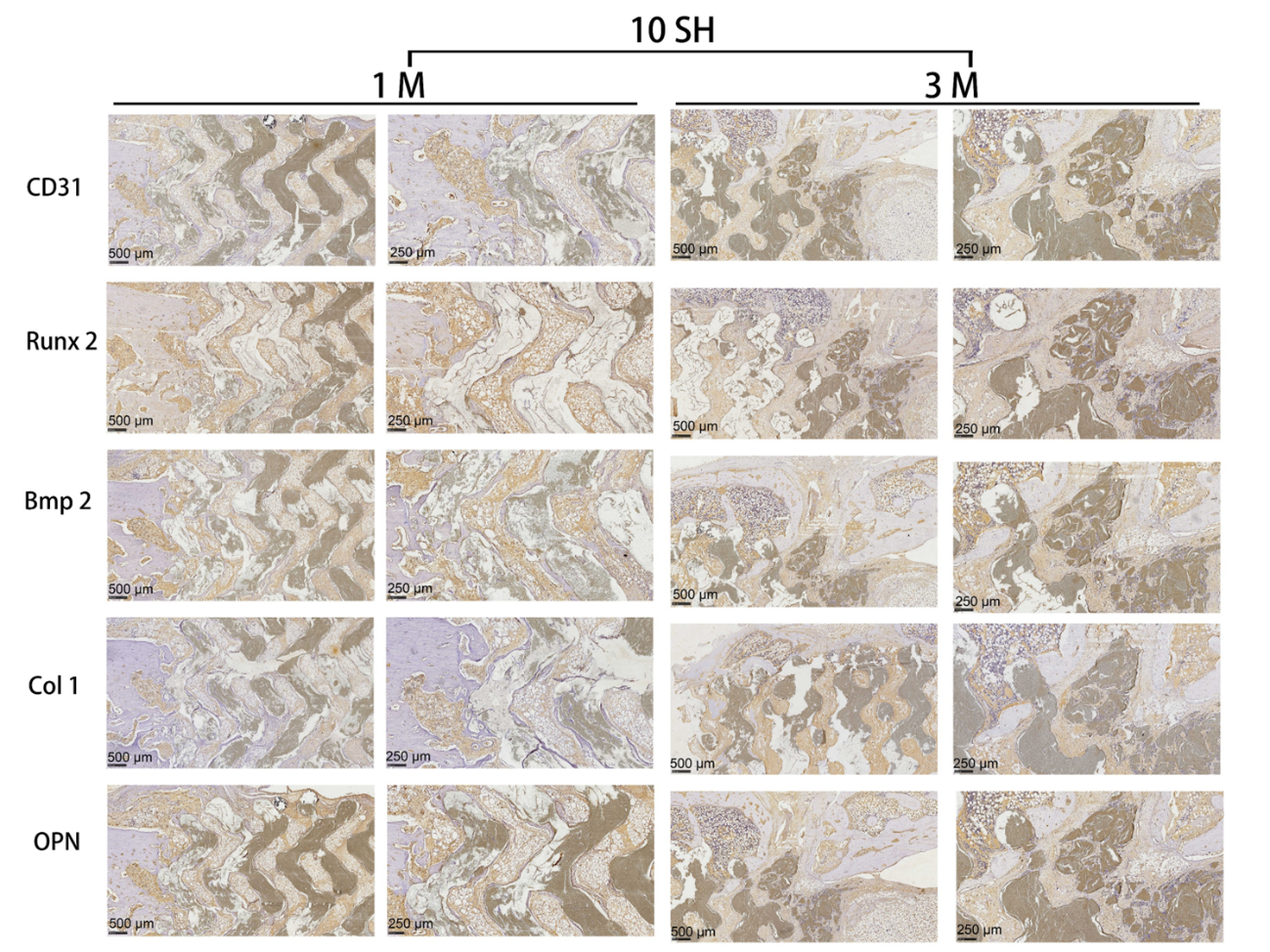


Fig. S14. Immunofluorescence staining of supercritical bone defect repair in 10SH. Mark the osteogenesis-related protein as CD31, Runx2, Bmp2, Col1, and Opn, among which the positive expression is brown.


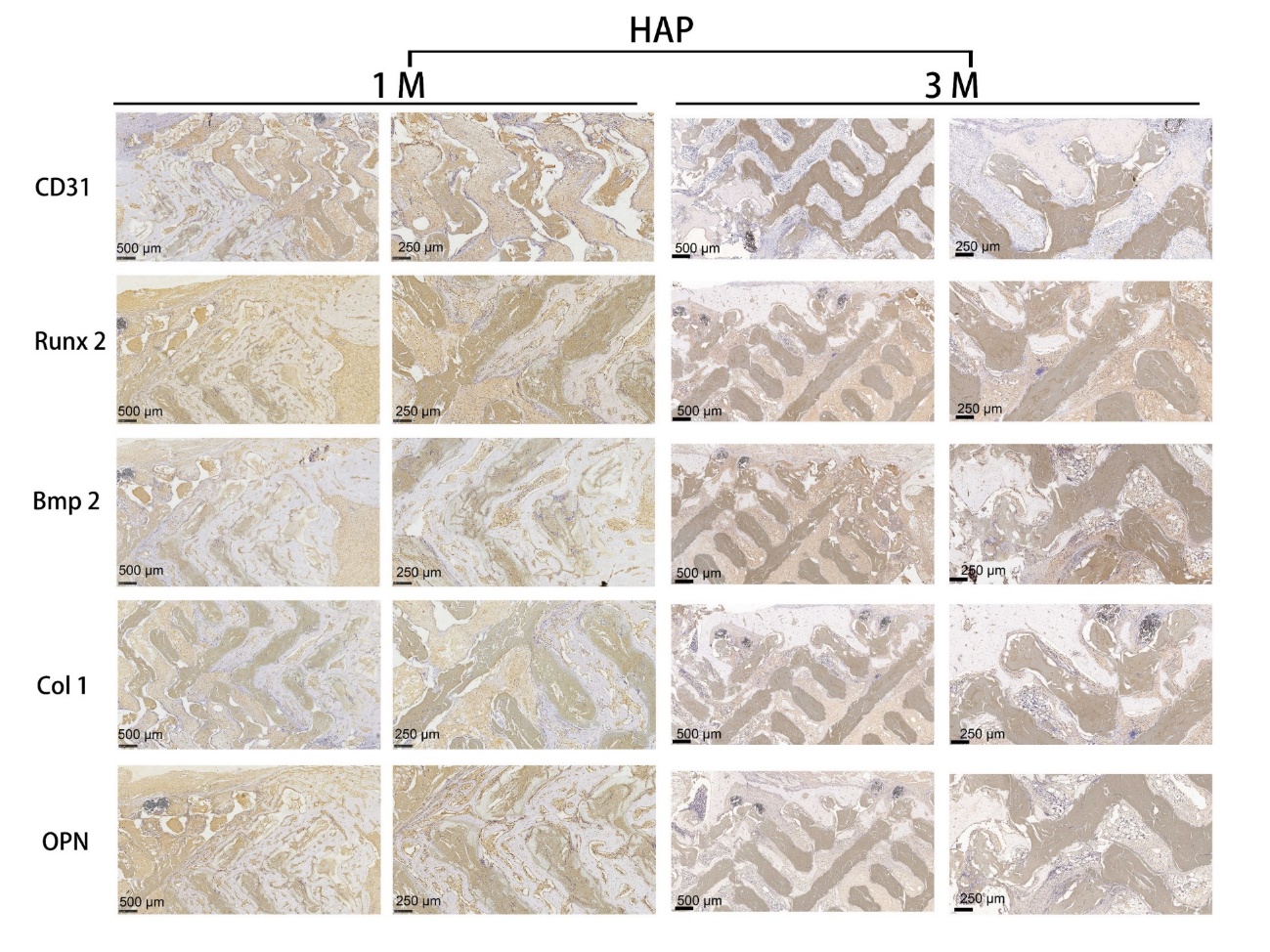


Fig. S15. Immunofluorescence staining of supercritical bone defect repair in HAP. Mark the osteogenesis-related protein as CD31, Runx2, Bmp2, Col1, and Opn, among which the positive expression is brown.


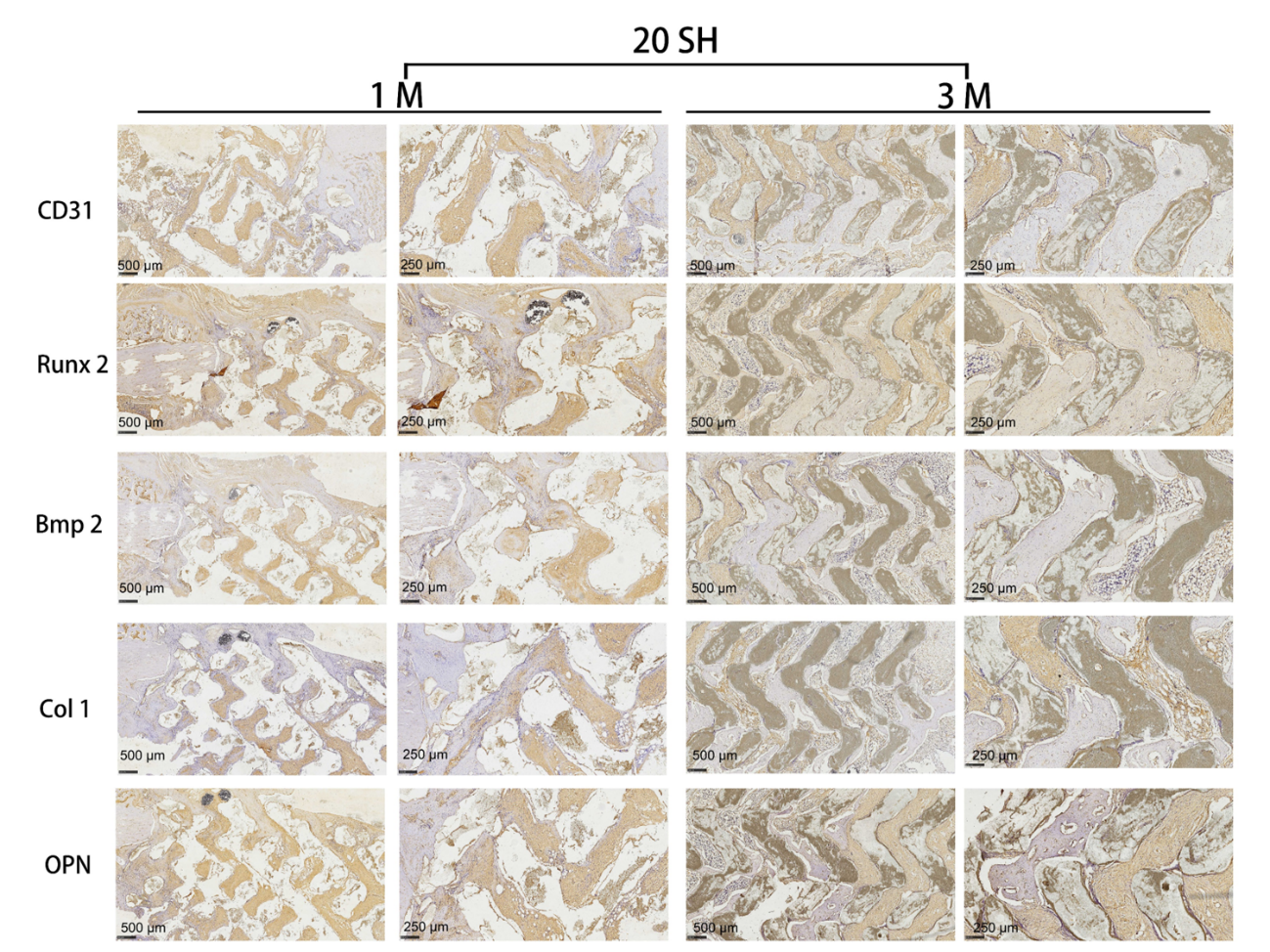


**Fig. S16. Immunofluorescence staining of supercritical bone defect repair in 20SH.** Mark the osteogenesis-related protein as CD31, Runx2, Bmp2, Col1, and Opn, among which the positive expression is brown.


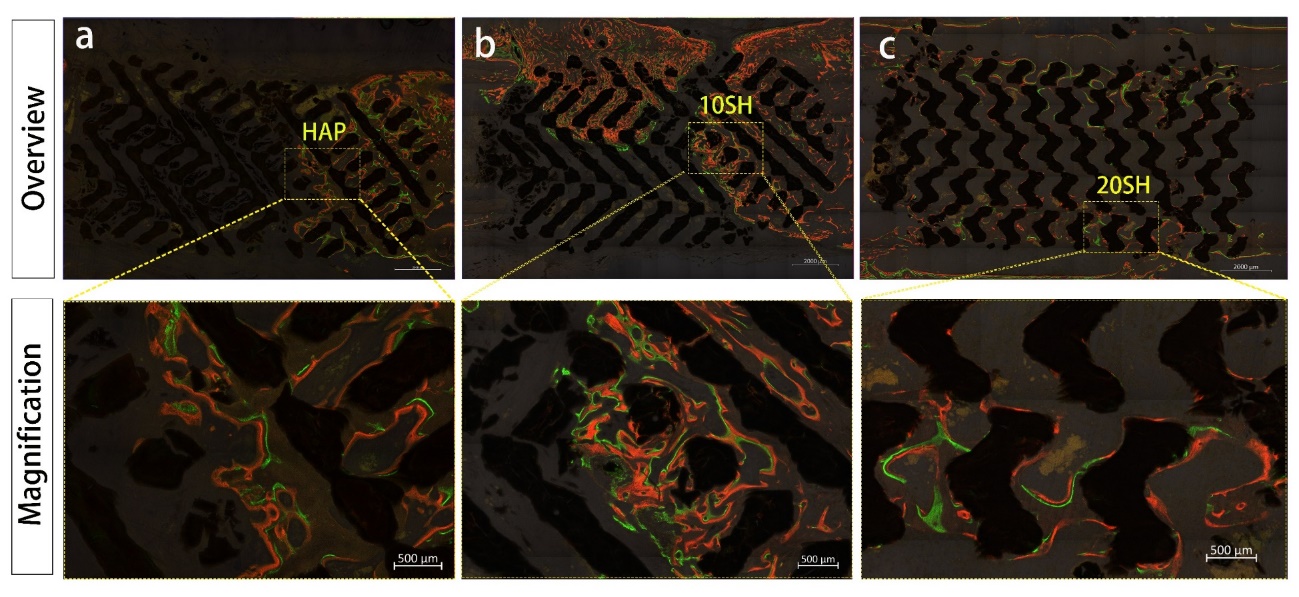


Fig. S17. Fluorescent labeling of new bone with calcein and retracycline hydrochloride. (a-c) Confocal laser scanning microscopy reconstructions of calcein and tetracycline labeling of mineral deposits, with the top row showing the overall morphology. The sections marked by yellow frames are magnified (orange-red for tetracycline labeling, green for calcein labeling).


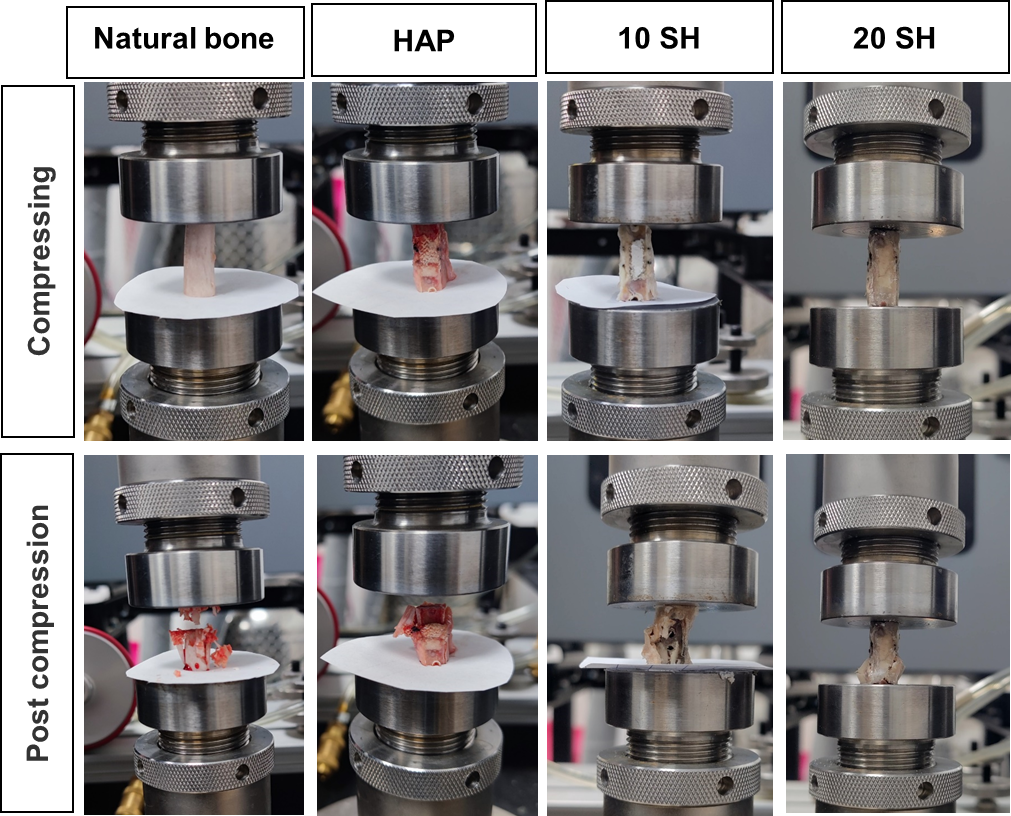


Fig. S18. After 3 months of implantation, the compression failure process of the IWRC.

Movie S1.

**Comparison of the Effects before and after the Reinforcement of the Porous Hydroxyapatite Ceramic.**
